# Flood resilience loci *SUBMERGENCE 1* and *ANAEROBIC GERMINATION 1* interact in seedlings established underwater

**DOI:** 10.1002/pld3.240

**Published:** 2020-07-21

**Authors:** Rejbana Alam, Maureen Hummel, Elaine Yeung, Anna M. Locke, John Carlos I. Ignacio, Miriam D. Baltazar, Zhenyu Jia, Abdelbagi M. Ismail, Endang M. Septiningsih, Julia Bailey‐Serres

**Affiliations:** ^1^ Department of Botany and Plant Sciences Center for Plant Cell Biology University of California Riverside Riverside CA USA; ^2^ International Rice Research Institute Metro Manila Philippines; ^3^ Department of Biological Sciences Cavite State University Indang Philippines; ^4^ Present address: Soybean and Nitrogen Fixation Research Unit USDA‐ARS Raleigh NC USA; ^5^ Present address: Department of Soil and Crop Sciences Texas A&M University College Station TX USA

**Keywords:** *AG1*, direct seeding, epistasis, *Oryza sativa*, pyramiding, *SUB1*, submergence tolerance

## Abstract

Crops with resilience to multiple climatic stresses are essential for increased yield stability. Here, we evaluate the interaction between two loci associated with flooding survival in rice (*Oryza sativa* L.). *ANAEROBIC GERMINATION 1* (*AG1*), encoding *trehalose 6‐phosphate phosphatase* 7 (*TPP7*), promotes mobilization of endosperm reserves to enhance the elongation of a hollow coleoptile in seeds that are seeded directly into shallow paddies. *SUBMERGENCE 1* (*SUB1*), encoding the ethylene‐responsive transcription factor *SUB1A‐1*, confers tolerance to complete submergence by dampening carbohydrate catabolism, to enhance recovery upon desubmergence. Interactions between *AG1/TPP7* and *SUB1/SUB1A‐1* were investigated under three flooding scenarios using four near‐isogenic lines by surveying growth and survival. Pyramiding of the two loci does not negatively affect anaerobic germination or vegetative‐stage submergence tolerance. However, the pyramided *AG1 SUB1* genotype displays reduced survival when seeds are planted underwater and maintained under submergence for 16 d. To better understand the roles of *TPP7* and *SUB1A‐1* and their interaction, temporal changes in carbohydrates and shoot transcriptomes were monitored in the four genotypes varying at the two loci at four developmental timeponts, from day 2 after seeding through day 14 of complete submergence. *TPP7* enhances early coleoptile elongation, whereas *SUB1A‐1* promotes precocious photoautotrophy and then restricts underwater elongation. By contrast, pyramiding of the *AG1* and *SUB1* slows elongation growth, the transition to photoautotrophy, and survival. mRNA‐sequencing highlights time‐dependent and genotype‐specific regulation of mRNAs associated with DNA repair, cell cycle, chromatin modification, plastid biogenesis, carbohydrate catabolism and transport, elongation growth, and other processes. These results suggest that interactions between *AG1/TPP7* and *SUB1/SUB1A‐1* could impact seedling establishment if paddy depth is not effectively managed after direct seeding.

## 1 INTRODUCTION

Rice (*Oryza sativa* L.) is unusual among crops for its ability to withstand flooding. Rice germplasm collections include accessions that can survive varied prolonged inundation scenarios, although some flooding survival strategies are absent in Green Revolution high‐yielding varieties, such as the widely grown *indica* cultivar IR64. There is a heightened need for cultivars with resilience to varied flooding scenarios because of climate change impacts on rain‐fed lowland ecosystems, comprising over 30% of international rice acreage (Bailey‐Serres et al., 2010). This need is most significant in regions where climate models predict increased frequency and severity of flooding events, such as in South and Southeast Asia (Hirabayashi et al., 2013).

Heavy rainfall or poor soil drainage can result in oxygen‐deficient soil (Greenway & Setter, 1996; Ismail, Ella, Vergara, & Mackill, 2009). Unusual among cereal crops, rice can germinate anaerobically in flooded soils by prioritizing the elongation of the coleoptile, a tubular embryonic first leaf, over the development and elongation of roots. This trait, known as anaerobic germination, can enable the hollow coleoptile to reach the aerated water surface, facilitating the oxygenation of seed tissues and promoting development (Angaji, Septiningsih, Mackill, & Ismail, 2009; Ella & Setter, 1999; Magneschi, Kudahettige, Alpi, & Perata, 2009; Taylor, 1942; Yamauchi, Aguilar, Vaughan, & Seshu, 1993). Anaerobic germination tolerance is agronomically advantageous, allowing the direct seeding of rice in paddies, reducing transplantation labor costs, water usage, and minimizing growth of flood‐intolerant weed species (Ismail et al., 2012; Septiningsih, Collard, et al., 2013; Tuong, Singh, Siopongco, & Wade, 2000). The quantitative trait locus (QTL) *ANAEROBIC GERMINATION 1* (*AG1*) was identified from the *japonica* landrace Khao Hlan On (Angaji et al., 2009) and defined as *TREHALOSE‐6‐PHOSPHATE PHOSPHATASE 7* (*TPP7*), encoding an enzyme that catalyzes the conversion of the low‐abundance metabolite trehalose‐6‐phosphate (T6P) to trehalose (Kretzschmar et al., 2015). T6P acts in the sensing of sucrose abundance, contributing to the control of catabolic carbon metabolism and the allocation of carbon from source to sink tissues (Figueroa & Lunn, 2016; Yadav et al., 2014). Elevated levels of T6P repress the activity of the catalytic subunit of the energy‐sensing kinase, SUCROSE NON‐FERMENTING‐1‐RELATED PROTEIN KINASE 1A (SnRK1A) (Zhang et al., 2009). Poor anaerobic germination of the high‐yielding variety IR64 is attributed to a chromosomal deletion that includes *TPP7* (Kretzschmar et al., 2015). The introduction of an ectopically‐expressed *TPP7* transgene into IR64 is sufficient to elevate trehalose in coleoptiles of seeds germinated underwater for 4 d, enhancing the activation of α‐*AMYLASE* (*AMY*) genes associated with endospermic starch catabolism, early elongation growth, and anaerobic germination tolerance (Kretzschmar et al., 2015). In *japonica* cultivars of rice possessing *TPP7,* additional genes contribute to elongation of the coleoptile during anaerobic germination (Nghi et al., 2019).

During anaerobic germination, *CBL‐INTERACTING PROTEIN KINASE 15* (*CIPK15*) is upregulated in embryo tissues in response to sugar starvation to promote SnRK1A activation, amplifying transcription of specific *AMY* genes in the scutellum, through which nutrients are transferred from the endosperm to the embryo (Lee et al., 2009; Lee, Chen, & Yu, 2014; Yu et al., 2015, Yu, Lee, Lo, & Ho, 2019). Nuclear localization of SnRK1A is required for its activation and is promoted by hypoxia and sucrose starvation (Ramon et al., 2019). Once activated, SnRK1A phosphorylates the transcription factor MYBS2 that represses *AMY3D* transcription. Phosphorylation promotes MYBS2 export to the cytoplasm, enabling nuclear‐localized MYBS1 to transactivate *AMY* genes (*i.e., AMY3D* and possibly *AMY3E*) via the same *cis*‐regulatory element as MYBS2 but at higher levels (Chen et al., 2019; Lee et al., 2014; Zhang et al., 2009). A third MYB, MYBGA, binds to a distinct motif in the *AMY3D* promoter and synergistically supports MYBS1 nuclear localization and *AMY3D* transcription (Hong et al., 2012). CIPK15 also contributes to the upregulation of *ALCOHOL DEHYDROGENASE* (*ADH*) (Lee et al., 2009), necessary to sustain NAD^+^ regeneration for anaerobic respiration. Coupling of SnRK1A‐dependent starch catabolism and anaerobic metabolism provides the energy to fuel underwater coleoptile elongation (Lee et al., 2014).

Rice plants often endure partial to complete submergence as a result of flash flooding and poor irrigation control. Submergence of photosynthetically active shoots entraps ethylene, which activates growth by elongation towards the water surface (Voesenek & Bailey‐Serres, 2015). Photosynthesis occurs in submerged rice through active gas exchange between surrounding floodwater and leaves if illumination, temperature, and dissolved CO_2_ and O_2_ conditions are favorable (Das, Panda, Sarkar, Reddy, & Ismail, 2009; Winkel, Colmer, Ismail, & Pedersen, 2013). The submergence escape strategy is effective if sufficient shoot tissue emerges from the inundation before photoassimilates are exhausted. An enhancement of complete submergence tolerance during vegetative development is provided by *SUBMERGENCE 1A‐1* (*SUB1A‐1*), identified as the submergene tolerance determinant within the *SUB1* QTL of the *aus* landrace FR13A (Fukao, Xu, Ronald, & Bailey‐Serres, 2006; Xu et al., 2006). By contrast to the effect of *TPP7* on early underwater coleoptile elongation*, SUB1A‐1* suppresses underwater elongation growth of seedlings after their transplantation into paddies (Septiningsih et al., 2009). SUB1A‐1 prolongs survival through quiescence of growth. This involves limiting starch catabolism (Fukao & Bailey‐Serres, 2008; Fukao et al., 2006) and promoting post‐submergence recovery (Alpuerto, Hussain, & Fukao, 2016; Fukao, Yeung, & Bailey‐Serres, 2011; Locke, Barding, Sathnur, Larive, & Bailey‐Serres, 2017).

*SUB1A‐1,* encoding an ethylene‐responsive transcription factor of subgroup VII (ERF‐VII), is upregulated by ethylene in submerged plants (Fukao et al., 2006), leading higher levels of two gibberellin (GA) signaling repressors, SLENDER LEAF 1 (SLR1) and SLENDER LEAF‐LIKE 1 (SLRL1) (Fukao & Bailey‐Serres, 2008). *SUB1A‐1* genotypes also alter brassinosteroid synthesis, elevating mRNAs associated with GA catabolism (Schmitz, Folsom, Jikamaru, Ronald, & Walia, 2013). As a result, SUB1A‐1 conditionally reduces GA‐responsiveness, dampening the upregulation of *AMY3D*, *AMY3E,* and *EXPANSIN* (*EXP*) mRNAs, the latter associated with anisotropic cell wall elongation in submerged shoot tissue (Fukao & Bailey‐Serres, 2008; Fukao et al., 2006; Jung et al., 2010; Locke et al., 2017; Mustroph et al., 2010). SUB1A‐1 promotes post‐submergence recovery by limiting reactive oxygen species production and accelerating the return to metabolic homeostasis (Alpuerto et al., 2016; Fukao et al., 2011; Locke et al., 2017). Recent studies show that *SUB1A‐1* transactivates genes encoding other ERF‐VIIs that, in turn, activate genes associated with submergence acclimation (Lin et al., 2019).

Molecular marker‐assisted introgression of *SUB1A‐1* into farmer‐preferred varieties including IR64, and cultivar adoption has increased the submergence tolerance of rice grown over 1.3 million hectares (Iftekharuddaula et al., 2011; Ismail, Singh, Singh, Dar, & Mackill, 2013; Mackill, Ismail, Singh, Labios, & Paris, 2012; Septiningsih, Collard, et al., 2013; Septiningsih et al., 2009, 2015; Septiningsih & Mackill, 2018; Singh et al., 2016). *AG1* has been introgressed into popular high‐yielding *indica* varieties, including IR64. Combining the flooding resilience conferred by *AG1/TTP7* and *SUB1/SUB1A‐1* by genetic pyramiding into farmer‐popular varieties could be advantageous to farmers. Indeed, genotypes carrying *AG1* and *SUB1* display anaerobic germination and vegetative‐stage submergence tolerance in small‐scale field trials (Toledo et al., 2015). Yet, the molecular consequences of genetic pyramiding of these two and other stress resilience loci are underexplored in crops.

Here, we investigated the interaction between *AG1* and *SUB1* loci, as their activities relative to submergence growth are contrasting, and their expression overlaps during submergence in the *japonica* cultivar M202(*SUB1*) (Jung et al., 2010; Mustroph et al., 2010). Four near‐isogenic genotypes differing at the *AG1* and *SUB1* loci were evaluated under three flooding scenarios experienced in rice plantations: (1) direct‐seeding in a shallow paddy to test anaerobic germination; (2) deep submergence of established seedlings to test vegetative‐stage submergence tolerance; and (3) direct‐seeding in a shallow paddy with submergence maintained for 16 days to test prolonged submergence tolerance from seeding through the transition to photoautotrophic growth. Neither anaerobic germination nor deep submergence tolerance was compromised in the pyramided genotype. Epistatic interactions between *AG1* and *SUB1* were evident in the third flooding scenario based on growth, survival, carbohydrates, and transcriptome analyses. Pyramiding of *AG1/TPP7* and *SUB1/SUB1A‐1* dampens early coleoptile elongation, delays the transition to photoautotrophy*,* and enhances the inhibition of elongation growth. This study predicts that the pyramided genotype may be less likely to thrive if submergence is prolonged after seeding, yet the IR64(*AG1,SUB1*) genotype should be effective in areas where shallow temporal flooding following direct seeding is well‐controlled, as shown by Chamara et al. (2018).

## 2 MATERIALS AND METHODS

### 2.1 Plant materials

Near‐isogenic lines in the IR64 cultivar were developed by introgression and bulked at the International Rice Research Institute (IRRI, Los Baños, Philippines). These included the genotypes IR64, IR64(*AG1*) (IR 93312‐30‐101‐20‐3‐66‐6), IR64*(SUB1*) (IR84194‐139), and IR64(*AG1,SUB1*) (IR 97703‐468‐21‐39) (Table S1a). The *SUB1A*, *SUB1C*, and *AG1/TPP7* alleles of these genotypes were confirmed by RNA‐sequencing (Figure S1). Additional IRRI lines included IR42 and the parental landraces KHO (Khao Hlan On) and FR13A (Flooding Resitant 13A). After postharvest drying, seeds were stored at 4°C and used within 3–18 months. To break dormancy, seeds were incubated at 50°C for 5 d. Plants for seed production were grown from the dry/winter season through the early wet season of 2013 at IRRI. Seeds for coleoptile and plumule length measurements were produced in 2012 in a greenhouse at the University of California, Riverside.

### 2.2 Survival evaluation of anaerobic germination and vegetative‐stage submergence

Seedling survival of underwater germinating seeds and shoot escape followed prior work (Septiningsih, Ignacio, et al., 2013) in biological triplicate. Experiments were performed in an alpha lattice design in an IRRI nethouse under ambient light (~11 hr photoperiod) and temperature. Thirty dry seeds of each genotype and parental controls were sown on trays with 1.5 cm soil and overlaid with 0.5 cm soil. Trays were submerged under 10 cm water measured from the soil surface with daily adjustment. Germination was scored based on a ≥1 mm coleoptile emergence. Survival was scored after 21 d of submergence. Control germination was performed in air.

Vegetative‐stage submergence survival was evaluated in cool months (December and February) at IRRI in biological triplicate following prior work (Septiningsih et al., 2012) in a randomized block design. Thirty seeds were dark‐germinated on moist paper towels for 2 d at 32°C and transplanted into the soil at 1 cm depth in plastic trays (54.0 × 38.5 × 9.6 cm), and placed in tanks in a nethouse. After 2 wk of growth, trays of seedlings at similar developmental age were submerged completely under 1.3–1.5 m water above the soil surface. Plants were desubmerged after 42 d, when IR42 control seedlings were visibly damaged. Control plants were grown in adjacent flats with standard watering. Survival was scored based on new tiller and leaf growth 21 d after desubmergence.

### 2.3 Combined anaerobic germination and vegetative‐stage submergence stress treatment

Triplicate biological experiments were performed under ambient temperature and ~12 hr photoperiod conditions in a nethouse at IRRI. Pots (14 × 9 × 9 cm) filled with 11 cm soil were covered with a cotton net (Figure S2a). One‐hundred dry seeds from IR64, IR64(*AG1*), IR64(*SUB1*), and IR64(*AG1,SUB1*) were placed onto the cotton net and covered with 0.5 cm soil. Pots were submerged in acrylic plastic aquaria (80 × 74 × 74 cm) 5 cm above the soil. Water was added daily to maintain 5–10 cm above the tallest shoot tip. Pots were desubmerged every other day from 2 to 14 d for photodocumentation, measurement, and tissue harvest. After 16 d, plants were desubmerged and allowed to recover under standard watering for 21 d before scoring new tiller development. Control plants were grown in air. At harvest time points, desubmerged whole seedlings (*n* = 25) were weighed to determine fresh weight biomass and then dried at 50°C for 5 d to determine dry weight biomass. From photographs, shoot length and germination (≥1 mm coleoptile emergence) were scored using ImageJ. Seeds that failed to germinate or had inadvertently broken coleoptiles were not scored. For carbohydrate analyses, endosperm‐scutellar‐embryo tissue of 30 desubmerged seedlings was harvested from 2 to 14 d, and coleoptile‐shoot tissues were harvested at 8, 10, and 12 d. Coleoptile‐shoot tissues of younger seedlings were not harvested because tissue quantity was insufficient for the analyses. Embryo‐coleoptile‐shoot tissue from 45 seedlings was harvested for RNA isolation. All tissues were harvested within 12 min of desubmergence and stored at −80°C until use. The seedling regions harvested for each assay are summarized in Figure S3a.

For anaerobic germination (underwater) in darkness, dehulled seeds were surface‐sterilized with 2.6% (v/v) sodium hypochlorite containing 0.02% (v/v) Tween‐20 for 10 min, rinsed, and transferred to a 250 ml beaker containing 200 ml (6.3 cm depth) ddH_2_O and covered with foil. For aerobic germination, 5.5 ml ddH_2_O was used, and the foil cover was punctured to allow aeration. Samples were placed at 23°C in darkness for 4 d. The percentage of dissolved oxygen in the ddH_2_O was measured at the start and end of the experiment with a fiber optic oxygen meter (Neofox Sport, Ocean Optics, Dunedin, USA), in five replicates. Oxygen content was 18.25% at the start (0 d) and 18.33% at the end (4 d) of the anaerobic germination test.

### 2.4 Carbohydrate quantification from endosperm and shoot tissues

Starch and soluble sugar content measurements followed (Ismail et al., 2009). Briefly, 50 mg of pulverized endosperm‐scutellar‐embryo or coleoptile‐shoot freeze‐dried tissue was extracted 3× in 80% (v/v) ethanol (2× in 7 ml and 1× in 5 ml ethanol) heated at 80°C for 15 min, vortexed, cooled to 25°C and centrifuged at 4,500 *g* for 10 min at 4°C. The insoluble pellet was used for starch analysis, whereas the combined supernatant was used for soluble sugar analysis. Sugar was measured using anthrone reagent (AR; 2.67:1 anthrone:ethanol). 3 ml AR was added to 300 µl of supernatant, vortexed, heated at 95°C for 10 min, cooled on ice, and equilibrated to 25°C before spectrophotometric reading at 620 nm. A standard curve was constructed with 0, 12.5, 25, 50, 100, and 200 µg glucose reacting with 3 ml AR. To measure starch, the insoluble pellet was dried at 70°C for 24 hr for dry weight and processed following Ismail et al. (2009). The dried starch (≤20 mg) was solubilized in 2 ml of acetate buffer (25 mM sodium acetate, pH 4.6) in a boiling water bath for 3 hr with stirring every 20 min. The solution was further hydrolyzed using 1 ml of acetate buffer containing 1 ml amyloglucosidase (0.38 mg unit per reaction; Sigma‐Aldrich A1602) at 37°C for 24 hr. Samples were centrifuged at 4,500 *g* for 10 min, and the supernatant was reserved. The pellet was washed with 3 ml of water and centrifuged to collect the supernatant again. The volume of combined supernatant was raised to 25 ml with water. The hydrolyzed starch supernatant was analyzed colorimetrically using a Peroxidase/Glucose Oxidase (P/GO) enzyme mixture (1:5 units peroxidase:glucose oxidase, Sigma‐Aldrich P7119) and a color reagent (o‐dianisidine dihydrochloride; Sigma‐Aldrich F5803), prepared by mixing a P/GO enzyme capsule (100 units) with 100 ml water and 4 mg color reagent (500 units). 600 µl supernatant was mixed with 3 ml P/GO‐color reagent and dark‐incubated at RT for 30 min before recording the absorbance at 450 nm. A standard curve was constructed with 0, 10, 20, 40, and 60 µg starch in 3 ml reagent. Three technical and three biological replicates were assayed for these metabolites.

### 2.5 Molecular genotyping of *AG1/TPP7* and *SUB1A*

The mapped RNA‐seq data were used to genotype these loci (Figure S1). *AG1* was confirmed by the absence of the interval containing *TPP7* on chromosome 9 (Kretzschmar et al., 2015). *SUB1A* and *SUB1C* genotyped by visualization of known nucleotide polymorphisms (Xu et al., 2006).

### 2.6 RNA extraction, RNA‐seq libraries construction, and sequencing

Figure S4 illustrates the transcriptomics pipeline from harvest through bioinformatic analyses. Total RNA was extracted from 0.5 ml of pulverized tissue by vortexing in 800 µl extraction buffer I [100 mM Tris (pH 8.0), 150 mM LiCl, 50 mM EDTA, 1.5% (w/v) SDS, 1.5% (v/v) 2‐mercaptoethanol] (Li & Trick, 2005). Then, 500 µl of 1:1 phenol:chloroform (pH 4.7) was added and centrifuged at 13,000 *g* for 15 min at 4°C. The upper aqueous phase was collected and mixed with 500 µl extraction buffer II [70% (w/v) guanidinium sulfate, 0.75 M sodium citrate, 10% (w/v) lauryl‐sarcosine, 2 M sodium acetate (pH 4.0)], incubated for 10 min at 25°C, mixed with 400 µl 24:1 chloroform:isoamyl alcohol, and centrifuged. The supernatant was mixed with 500 µl isopropanol and 400 µl 1.2 M sodium chloride, incubated on ice for 15 min, and then centrifuged. RNA pellets were washed with 800 µl 70% (v/v) ethanol, dried for 15–20 min at 25°C, resuspended in 30 µl RNase‐free water, and stored at −80°C until further use. RNA quality was checked by agarose gel electrophoresis and quantified with a NanoDrop ND‐1000 spectrophotometer.

RNA‐Seq libraries were constructed following the high‐throughput Illumina strand‐specific RNA sequencing library protocol from Wang et al. (2011). Libraries had the expected fragment size (∼250 bp) with little or no primer contamination, based on agarose gel electrophoresis and Bioanalyzer (2100 Expert High Sensitivity DNA Assay) results. Library yield and concentration were evaluated with a Qubit 2.0 Fluorometer (Life Technologies). Index primers were used to amplify libraries (Table S1b). Paired‐end sequencing (2 × 50 cycles) was performed on the Illumina HiSeq 2500 by the Genomics Core Facility of the Institute for Integrative Genome Biology, UC Riverside.

### 2.7 Bioinformatic and statistical analyses

The flow diagram of the analyses is shown in Figure S4. Fastq files were processed by trimming with trim_galore (Babraham Bioinformatics, version 0.4.1). Sequencing reads overlapping with the adapter (Table S1c) for a minimum of 10 bp were trimmed using the full‐length adapters in paired‐end mode. Reads < 25 bp after trimming were discarded. Trimmed reads were aligned using Bowtie2 (version 2.2.5) and Tophat (version 2.0.14) to the *japonica* Nipponbare genome (IRGSP‐1.0.30 Ensembl) to which the *SUB1A‐1* gene sequence (DQ011598.1; Xu et al., 2006) was added. The Nipponbare genome encodes TPP7. Mapped reads were counted on exons using the “union mode.” Counting was performed using the GenomicFeatures and Rtracklayer packages. All bioinformatic analyses, except trimming, were done using R with the SystemPipeR workflow (Backman & Girke, 2016). Alignment statistics are listed in Dataset S1a. Libraries were prepared and sequenced in triplicate, and except IR64(*AG1,SUB1*) at 2 and 4 d, and IR64(*SUB1*) at 8 d, for which only two of three replicates were successfully sequenced.

RNA‐seq count data were analyzed using the edgeR package in R. The raw read count data were offset by adding five counts to each transcript isoform, so those with very low transcript count data could be included as genes in fold change analyses. The offset data were then normalized by the trimmed mean of M‐values (TMM) normalization in edgeR and analyzed to estimate dispersions (common, trended, and tagwise) and fitting to a negative binomial model. To determine differentially expressed genes (DEGs), the generalized linear model (GLM) likelihood ratio test was used in edgeR.

Linear regression model and ANOVA functions implemented in R (stats package) were used to analyze the log_2_ CPM (count per million read) data generated with edgeR. Genotypes and days of data collection were treated as categorical variables, and the main effects and interactions for these two variables were estimated and tested for each transcript. The Benjamini‐Hochberg Procedure was used to adjust the p‐values, providing a false discovery rate (FDR). DEGs (log_2_ FC > |1| and FDR < 0.05) were compared between genotypes on specific days (genotypic comparisons) or between days in specific genotypes (temporal comparisons) by Partitioning Around Medoids (PAM) clustering using FC values (FC Cluster Analysis) and log_2_ counts per million read (CPM) values (CPM Cluster Analysis). The BioMart database (‘plants.ensembl.org’ > ‘plants_mart’ > ‘osativa_eg_gene’) was used to obtain the GO terms (‘go_accession’ and ‘go_namespace_1006’).

### 2.8 Quantitative real‐time reverse transcriptase polymerase chain reaction (qRT‐PCR)

Total RNA was extracted with an RNeasy Plant Mini Kit (Qiagen). cDNA synthesis and qRT‐PCR were performed following (Juntawong, Girke, Bazin, & Bailey‐Serres, 2014). LOC_Os01g53520 was used for normalization. Primer information is in Table S1c.

### 2.9 Data deposition

The transcriptomic data that support the findings of this study are openly available in the NCBI GEO database (accession no. GSE136885).

## 3 RESULTS AND DISCUSSION

### 3.1 Anaerobic germination enhanced by *AG1* is not altered by the presence of *SUB1*

**FIGURE 1:**
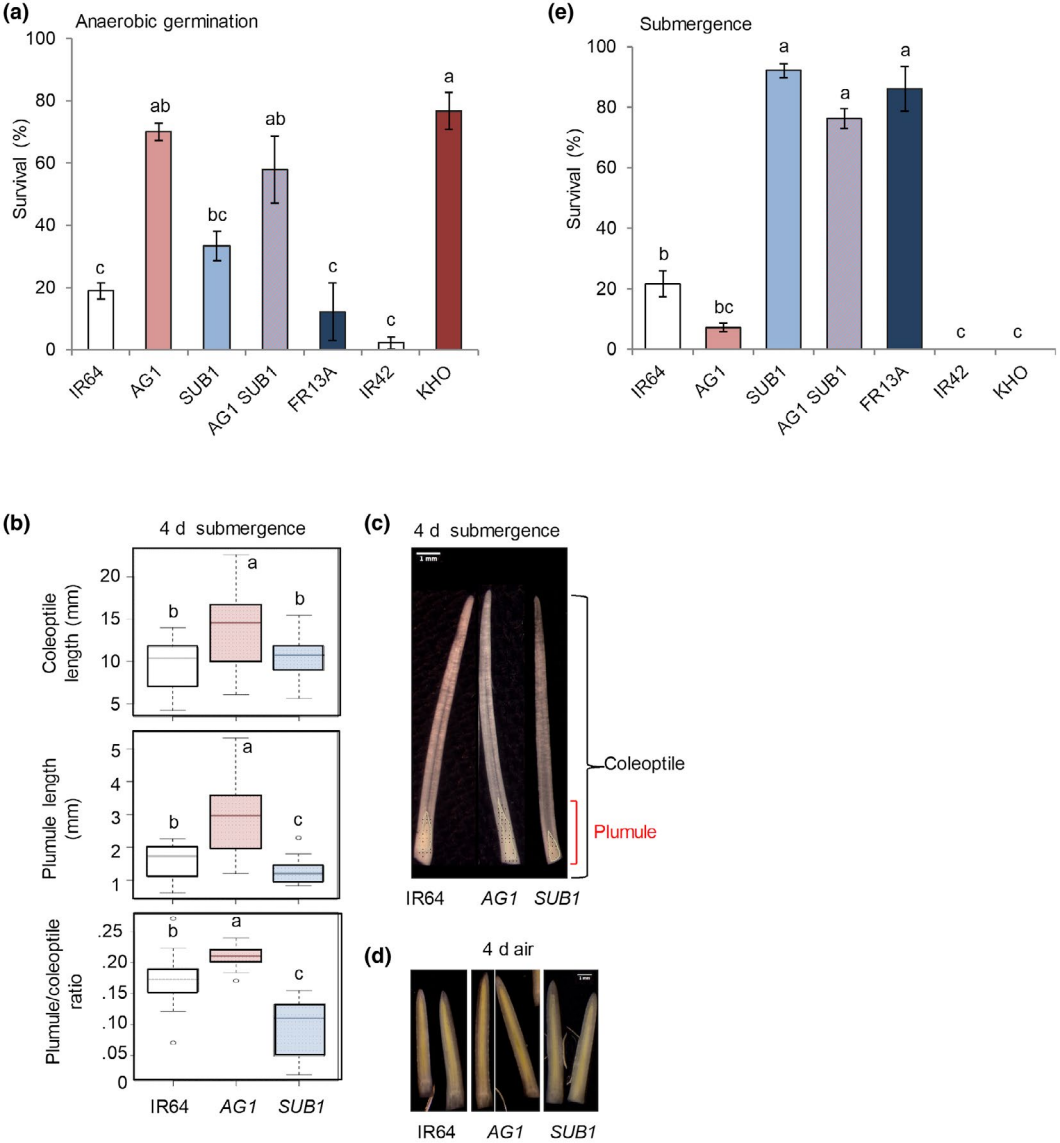
Pyramiding of *AG1* and *SUB1* in IR64 maintains anaerobic germination tolerance and vegetative‐stage submergence tolerance under respective submergence conditions. (a) *AG1* enhanced plant survival under anaerobic germination in genetic backgrounds with or without *SUB1*. This assay was performed in the soil by dry seeding under ambient light and temperature in a nethouse. IR64, IR64(*AG1*), IR64(*SUB1*), IR64(*AG1,SUB1*), FR13A, IR42, and KHO were dry seeded in soil and submerged at 10 cm water depth maintained for 21 d. Survival was scored if the shoot emerged above the water surface by day 21. (b–d) A hollow coleoptile develops from seeds grown under water. IR64, IR64(*AG1*), and IR64(*SUB1*) seeds were germinated in air or underwater (H_2_O) in complete darkness. After 4 d of growth, the length of the dissected coleoptile and plumule (leaf whorl surrounding the shoot apical meristem) were measured, and their was ratio calculated. Lower and upper whiskers indicate the minima and maxima, respectively; the box represents the interquartile range; the line in the box shows the median length from 3 independent biological replicates of 10 dissections. Circles indicate outliers. Dissected coleoptiles were observed with visible light at 5× magnification from underwater‐grown (b) and air‐grown (c) seeds. Plumules are outlined to enhance visualization. White bars indicate 1 mm. (e) *SUB1* maintained seedling survival of vegetative‐stage submergence in IR64(*AG1,SUB1*). 14‐d‐old seedlings of the seven genotypes were completely submerged at 1.3–1.5 m depth and desubmerged after 42 d when the majority of the IR42 plants displayed leaf death. Survival was confirmed as new tiller and leaf growth after 21 d of recovery. The data in panel (a) and (e) represent mean ± *SE* of 3 biological replicates (*n* = 30 seedlings of each genotype per replicate). Genotypes, indicated as *AG1*, *SUB1,* and *AG1 SUB1,* are in the IR64 background. Letters indicate significant differences (*p* < 0.05, ANOVA with Tukey HSD test)

To investigate if enhanced anaerobic germination conferred by *AG1/TPP7* is influenced by *SUB1/SUB1A‐1*, near‐isogenic IR64 lines varying at *AG1* and *SUB1*, and parental accessions were dry seeded in soil under a column of water. The *AG1* donor KHO and both IR64 lines homozygous for *AG1* survived direct seeding at significantly higher frequencies than IR64 (Figure 1a). Survival rates for IR64(*AG1*; 70%) and IR64(*AG1,SUB1*; 58%) were statistically indistinguishable. IR64(*SUB1*) had higher survivability (33.3%) than IR64 (18.9% survival), although the difference was insignificant. Survivability was poor for the inbred IR42 (2.2%), a reference genotype for anaerobic germination intolerance (Angaji et al., 2009), and the *SUB1* donor FR13A (12.2%), as reported previously (Ismail et al., 2009). FR13A is unrelated to IR64 and contains QTL in addition to *SUB1* that contribute to submergence tolerance (Gonzaga, Carandang, Sanchez, Mackill, & Septiningsih, 2016). We conclude that pyramiding of *TPP7* and *SUB1A‐1* in IR64 does not impact establishment by direct‐seeding in shallow floodwaters.

Prior studies monitored germination and shoot development during underwater germination in darkness (Kretzschmar et al., 2015; Narsai et al., 2015) or low oxygen conditions (Lasanthi‐Kudahettige et al., 2007; Magneschi et al., 2009) for short durations to compare genotypes. Thus, we evaluated the length of the coleoptile and plumule (shoot meristem region and surrounding leaflets within the coleoptile sheath) after germination in air or underwater in complete darkness. The hollow “snorkel” coleoptile and plumule of IR64(*AG1*) was longer than that of IR64 and IR64(*SUB1*) after 4 d of dark submergence (Figure 1b,c). Germination in air produced a leaf whorl filling the entire coleoptile at 4 d, which split by 5 d to allow for the emergence of the expanding shoot (Figure 1d), confirming that the snorkel phenotype is conditional to anaerobic germination. The IR64(*AG1*) characteristics observed are consistent with those reported by Kretzschmar et al. (2015).

### 3.2 Submergence tolerance conferred by *SUB1* is not compromised by *AG1*

To evaluate if vegetative‐stage submergence tolerance provided by *SUB1* is influenced by *AG1*, we measured the submergence survival of the four genotypes. Sixteen‐d‐old plants were fully submerged until leaves of the highly submergence‐sensitive genotype IR42 had deteriorated. *SUB1*‐containing lines had significantly higher survival rates (76%–92%) than the other genotypes (Figure 1e). As anticipated, non‐*SUB1* lines had poor survivability: IR64 (21.6%) and IR64(*AG1*) (7.1%). None of the IR42 or KHO plants recovered from the prolonged submergence. This demonstrates that *SUB1A‐1* confers submergence tolerance in the presence of *TPP7*, which is consistent with the knowledge that the *japonica* M202(*SUB1*) cultivar that encodes *SUB1A‐1* and *TPP7*, is tolerant to submergence (Fukao et al., 2006). Thus, pyramiding *AG1* with *SUB1* does not compromise the submergence resilience contributed by *SUB1A‐1* to vegetative stage seedlings.

### 3.3 *AG1* and *SUB1* interact when direct‐seeding is followed by extended submergence

**FIGURE 2:**
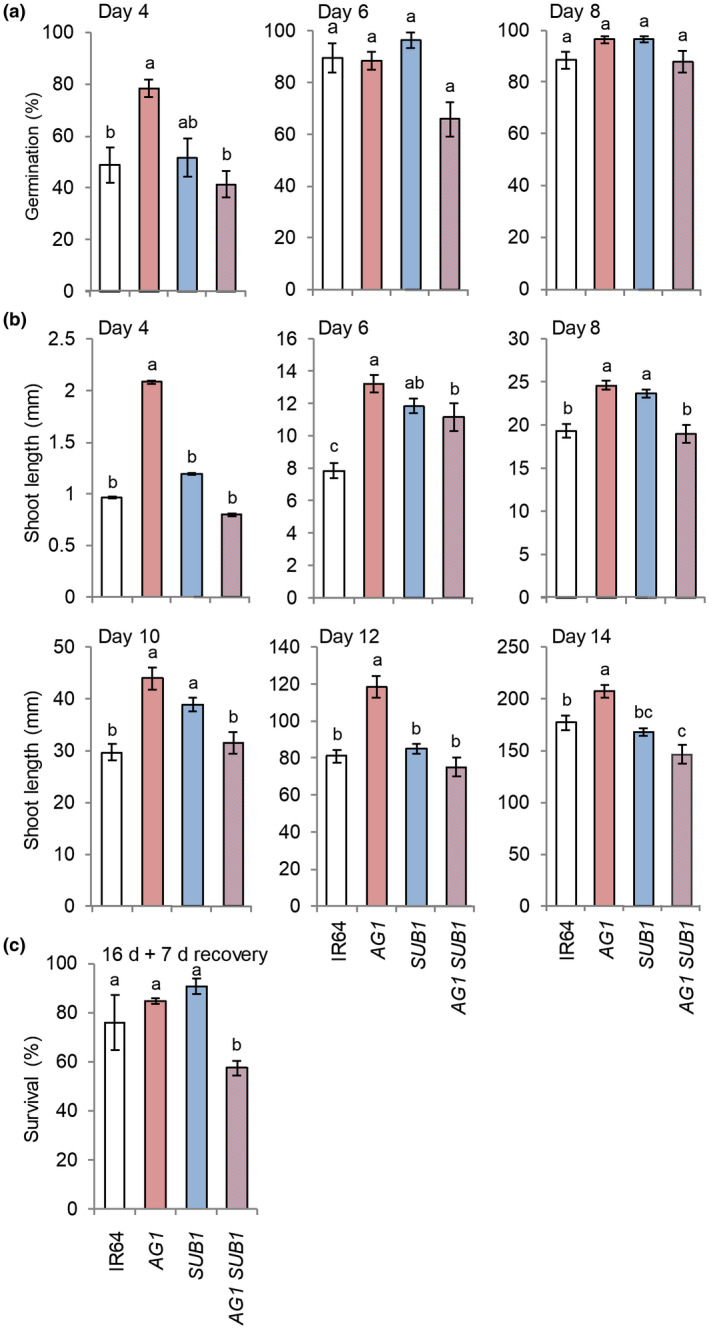
Germination, shoot growth, and survival of near‐isogenic lines, continually submerged up to 16 d, indicate *AG1* and *SUB1* interactions. IR64, IR64(*AG1*), IR64(*SUB1*), and IR64(*AG1,SUB1*) were dry seeded in soil and submerged completely with incremental addition of water for up to 16 d. (a) Percentage of germinated seed (coleoptile emergence ≥ 1 mm) at 4, 6, and 8 d of submergence. (b) Coleoptile/shoot length at 4, 6, 8, 10, 12, and 14 d of submergence. (c) Percentage of plants surviving 16 d of submergence, based on tiller and leaf growth, scored 7 d after desubmergence. Data represent mean ± *SE* of three biological replicates (*n* = 25–30 seedlings of each genotype per replicate). Letters represent significant differences (*p* < 0.05, ANOVA with Tukey HSD test)

Kretzschmar et al. (2015) demonstrated that the *TPP7* promoter is active in the aleurone, scutellum/embryo axis, coleoptile, and root of seeds after 2 and 4 d of germination underwater in darkness. Data of other studies show that *SUB1A‐1* mRNA accumulates in shoots of 14‐d‐old plants within one hour of submergence and is maintained for more than two weeks (Fukao et al., 2006; Xu et al., 2006) and *TPP7* mRNA accumulates during submergence in both near isogenic cultivars varying at *SUB1A* (Jung et al., 2010; Mustroph et al., 2010). Given the opposing roles of these genes in underwater growth at the germination and vegetative stage, respectively, an interaction between loci may occur in underwater‐seeded rice that is unable to emerge into the air before exhaustion of seed reserves or transitioning to photoautotrophy. To evaluate this hypothesis, we dry seeded and maintained the four genotypes under complete submergence for 16 d (Figure S2a,d). None of the genotypes had germinated after 2 d underwater, IR64(*AG1*) had the highest percentage of germinated seeds (78%) after 4 d, all genotypes except IR64(*AG1,SUB1*) matched IR64(*AG1*) germination after 6 d, and all genotypes approached 100% germination after 8 d (Figure 2a).

To evaluate genotypic control of shoot elongation, we measured the length of the coleoptile and emergent shoot from day 4 through 14 of submergence (Figure 2b; Figure S2). Differences in temporal elongation were observed between genotypes. The rapid coleoptile elongation conferred by *TPP7* was clear at 4 d for IR64(*AG1*) but conspicuously absent in IR64(*AG1,SUB1*). IR64, IR64(*SUB1*), and IR64(*AG1,SUB1*) had comparable coleoptile lengths at 4 d. After 6 d, etiolated coleoptiles of all genotypes extended from the soil, with no emergent leaves (Figure S2c). IR64(*SUB1*) and IR64(*AG1,SUB1*) had similar coleoptile/shoot length, whereas that of IR64 was significantly shorter. Coleoptiles split, the first true leaves emerged, and greening became evident after day 6. A similar number of green shoots was visible for IR64(*AG1*) and IR64(*SUB1*) at 8 d, with fewer visible for the other two genotypes. This transition was marked by a near‐doubling in shoot length every 2 d in all genotypes. IR64(*AG1*) shoots were consistently the most elongated, whereas elongation of IR64(*SUB1*) and IR64(*AG1,SUB1*) slowed between 12 and 14 d (Figure 2b; Figure S2d), characteristic of dampened underwater elongation conferred by *SUB1A‐1* (Fukao et al., 2006).

The recovery of new tillers after prolonged submergence (16 d) was the most compromised in IR64(*AG1,SUB1*) of the four near‐isogenic lines (Figure 2c). The percentage of seedling survival of the pyramided genotype (57%) was statistically distinguishable from IR64 (76%), IR64(*AG1*) (85%), and IR64(*SUB1*) (91%). This sensitivity contrasts with the vegetative‐stage submergence tolerance (Figure 1e) observed when submegence began after the transition to photoautotrophic growth. Following desubmergence, leaves of IR64(*SUB1*) were the most and IR64(*AG1,SUB1*) the least erect (Figure S2e), indicative of weakened cell wall rigidity associated with underwater elongation (Vreeburg et al., 2005). Survival of transient submergence is associated with changes in metabolism and growth that occur during submergence and post‐submergence recovery period (Fukao et al., 2011; Yeung, Bailey‐Serres, & Sasidharan, 2019; Yeung et al., 2018). The presence of *SUB1A‐1* dampens post‐submergence stresses that include a rapid burst in ROS and leaf dehydration (Fukao et al., 2011), it also accelerates recovery of carbon and nitrogen de‐subhomeostasis following desubmergence (Locke et al., 2017). Pyramiding *TPP7* with *SUB1A‐1* may have a negative consequence on these post‐submergence events. Taken together, these data demonstrate a genetic interaction between *TPP7* and *SUB1A‐1* that is of negative consequence if submergence at seeding continues through the transition to photoautotrophic development.

### 3.4 *AG1* and *SUB1* similarly influence biomass, soluble sugars, and starch of seed and shoot tissues during prolonged submergence

Based on prior observation that the embryo‐coleoptile region of dark‐grown (4 d) seeds of IR64(*AG1*) and transgenic IR64(*promoterTPP7:TPP7*) elevate α‐amylase activity and sucrose content relative to IR64 (Kretzschmar et al., 2015), we monitored nonhydrolyzable starch and soluble sugar contents over the 14 d submergence time course in endosperm and shoot tissue (Figure S3a–e). We also measured changes in whole seedling biomass. Seedling fresh weight (Figure S3f) increased after 10 d of submergence as shoot tissues elongated and expanded (Figure 2b), becoming significantly higher in IR64(*SUB1*) and lower in IR64(*AG1,SUB1*) by 14 d, relative to IR64. By contrast, seedling dry weight and the soluble sugar and starch contents of the endosperm were statistically indistinguishable between genotypes (Figure S3b,d,g), with the exception of a significant elevation of endospermic soluble sugar content in IR64(*SUB1*) at 12 d. The endosperm of IR64(*AG1*) and IR64(*SUB1*) tended to have lower starch content at later time points. Endospermic starch and sugar levels were progressive lower and higher, respectively, from 2 to 14 d underwater in all genotypes, indicating that seed reserves are tapped but not fully consumed during underwater seedling establishment, even after 14 d under submergence.

We also monitored sugar and starch content of shoots from 8 to 14 d (Figure S3c,e), commensurate with the greening and rapid elongation phase of underwater seedling development. Genotypes had statistically indistinguishable levels of these carbohydrates, except for modestly higher soluble sugar in IR64(*SUB1*) and IR64(*AG1,SUB1*) at 8 d. The presence of shoot starch in these illuminated plants is evidence of underwater photosynthesis. Thus, all four genotypes progressively mobilized endosperm reserves and transitioned to the production of photosynthate when maintained under constant submergence for 14 d following seeding underwater. Despite limited differences in starch and sugar metabolites, the distinction in coleoptile and shoot elongation dynamics and survival in these four genotypes motivated us to examine interactions between *TTP7* and *SUB1A‐1* using transcriptomics.

### 3.5 Transcriptomic survey of near‐isogenic genotypes differing at *AG1* and *SUB1*

A transcriptome analysis was performed with the same tissue used for the growth and metabolite study to identify transcripts regulated over developmental time by *AG1*/*TPP7* or *SUB1*/*SUB1A‐1* or as a result of their genetic interaction. Transcriptomes were sequenced at four time points: at 2 d after initiation of germination; at 4 d, when *AG1* enhanced coleoptile elongation in IR64(*AG1*); at 8 d, when leaves emerged from the senescencing coleoptile and shoot length doubled every 48 hr; and at 14 d, when a *SUB1A‐1* effect on shoot elongation was evident. Figure S4 illustrates the flow chart of mRNA‐sequencing and transcriptome analysis. RNA‐sequencing libraries produced 33 to 66 million reads, of which 95% mapped uniquely to exons (Dataset S1a).

As a first step, we compared *TPP7* and *SUB1A* transcript abundance determined by RNA‐sequencing over the developmental time course (2, 4, 8, and 14 d). qRT‐PCR validation was performed with additional sampling at 6, 10, and 12 d (Figure S5a–d). The sequence differences at *TPP7* and *SUB1A* of the four genotypes are shown in Figure S1a–d. As mentioned, IR64 lacks *TPP7* due to a deletion. IR64(*SUB1*) encodes *SUB1A‐1*, whereas IR64 encodes *SUB1A‐2*. Submergence tolerance is associated with pronounced induction of *SUB1A‐1,* whereas intolerance is correlated with limited upregulation of *SUB1A‐2* (Fukao et al., 2006; Iftekharuddaula et al., 2016; Sharma et al., 2018; Singh et al., 2010; Xu et al., 2006). Tolerance also correlates with a nucleotide polymorphism that distinguishes *SUB1A‐1* and *SUB1A‐*2 (Xu et al., 2006), conferring a serine residue in SUB1A‐1 that can be phosphorylated in vitro by mitogen‐activated protein kinase 3 (Singh & Sinha, 2016). *SUB1A‐1* transcripts were at the limit of detection in coleoptiles harvested at 2 and 4 d and increased between 4 and 14 d in IR64(*SUB1*) and IR64(*AG1,SUB1*) (Figure S5a). *SUB1A‐2* mRNA increased, although insignificantly, between 8 and 14 d in IR64 and IR64(*AG1*)*,* with maximum levels significantly lower than that of *SUB1A‐1* at both time points. *TPP7* mRNA was low at day 2 and rose significantly by 8 d in IR64(*AG1*) and IR64(*AG1,SUB1*) and was undetectable due to a deletion in IR64 and IR64(*SUB1*) (Figures S1b and S5b), validating the absence of this gene. As *SUB1A‐1* and *TPP7* are co‐expressed after 6 d (Figure S5c,d), we hypothesized that *SUB1A‐1* would have a limited impact on the transcriptome before day 4 of development underwater.

### 3.6 Transcriptome variations of the pyramided genotype demonstrate epigenetic interaction between *TPP7* and *SUB1A‐1*

**FIGURE 3:**
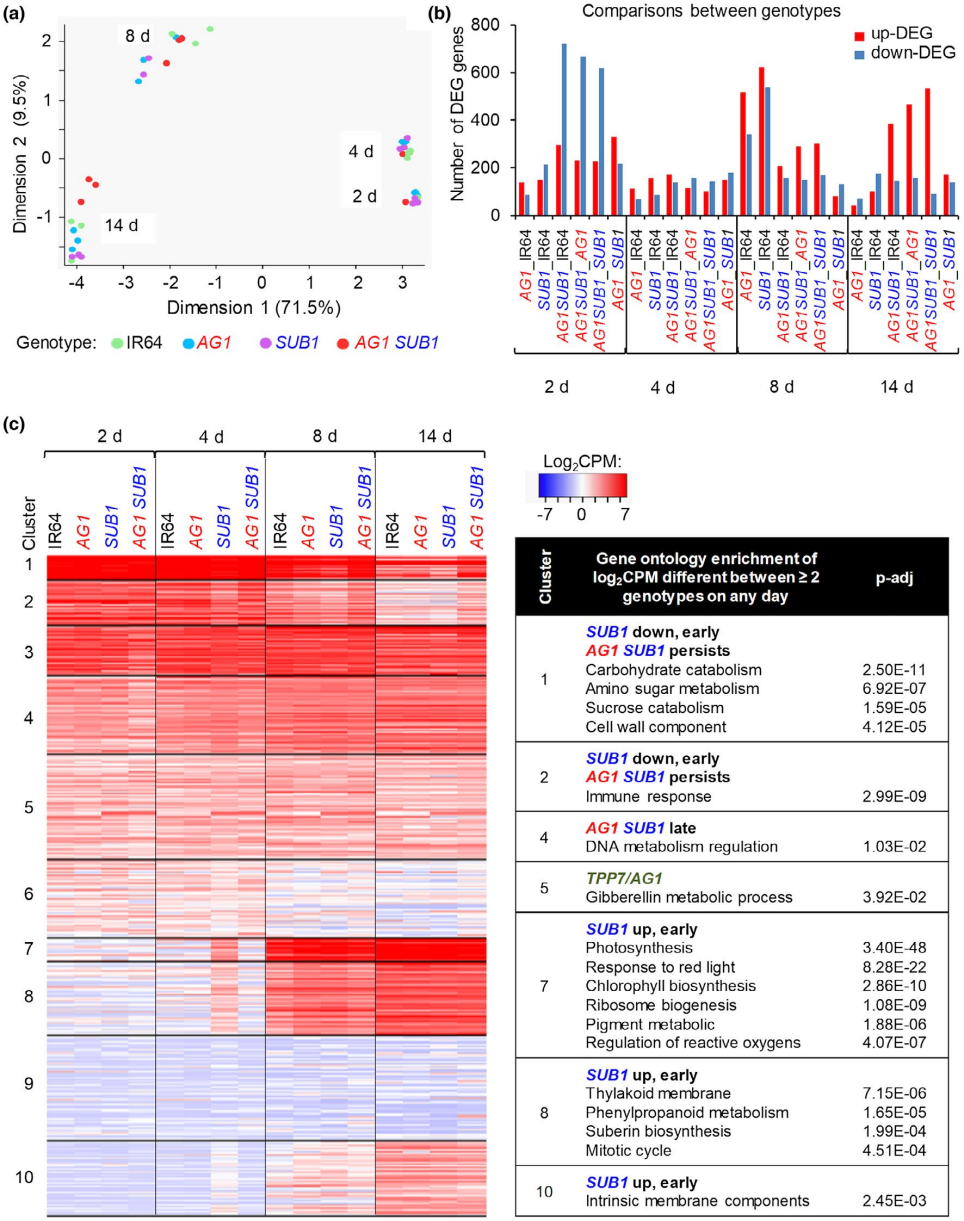
Transcriptomic changes during germination and seedling establishment of genotypes grown under prolonged submergence. (a) MDS analysis with dimensions 1 and 2 of transcriptomes from four time points and four genotypes determined using mean log_2_CPM values. (b) Differentially expressed gene (DEG; log_2_ FC > |1|; FDR < 0.05) numbers based on genotypic comparisons of log_2_CPM values over four submergence time points. (c) Cluster analyses (*n* = 10) of DEGs using mean log_2_CPM values of genotypes over four submergence time points. Representative Gene Ontology (GO) categories reflect analyses of key clusters for the degree of *AG1* and *SUB1* loci regulation. Genes and corresponding log_2_CPM values and GO terms in each cluster are in Dataset S1d, e

A multidimensional scaling (MDS) analysis was performed to gain an overall perspective of how the 16 transcriptomes varied by genotype and time. This revealed moderate genotypic differences and pronounced temporal variation over the submergence time course (Figure 3a). All genotypes grouped together at each time point, with the similar 2 and 4 d transcriptomes grouping separately from the 8 and 14 d transcriptomes on Dimension 1 (71.5% of the total variation). The 8 and 14 d samples separated on both Dimension 1 and Dimension 2 (9.5% of the total variation). This indicates that a major transcriptome transition occurs between 4 and 8 d that is followed by changes between 8 and 14 d. Transcriptomes of genotypes were most clearly separated at 14 d. Next, we identified differentially expressed genes (DEGs; FC [fold change] > |2|; FDR < 0.05) based on comparisons between genotypes and time points (Dataset S1). The pair‐wise comparison of DEG numbers between genotypes at each time point illustrates that IR64(*AG1,SUB1*) is the most distinct genotype (Figure 3b). This was most evident early (at 2 d) and late (at 14 d) when several hundred transcripts were dampened or elevated relative to the three other genotypes respectively.

To better view the interactions between *TPP7* and *SUB1A‐1*, we displayed the log_2_CPM values of DEGs between genotypes at each time point (Figure 3c; Dataset S1c,d,e), again exposing a transcriptome transition between 4 and 8 d. Based on Gene Ontology (GO) enrichment, days 2 and 4 represent a seed germination phase characterized by transcripts associated with seed nutrient reserve mobilization and cellular wall development (cluster 1). IR64(*SUB1*) was the first and IR64(*AG1,SUB1*) the last to downregulate these transcripts. The 4 to 8 d transition was marked by the upregulation of genes associated with plastid biogenesis, photosynthesis, and mitotic cell cycle genes (clusters 7, 8, and 10). This reconfiguration of the transcriptome coincides with the emergence and greening of shoot tissue by 8 d (Figure S2c). IR64(*SUB1*) displayed this transition early (4 d), followed by IR64(*AG1*) at 8 d.

**FIGURE 4:**
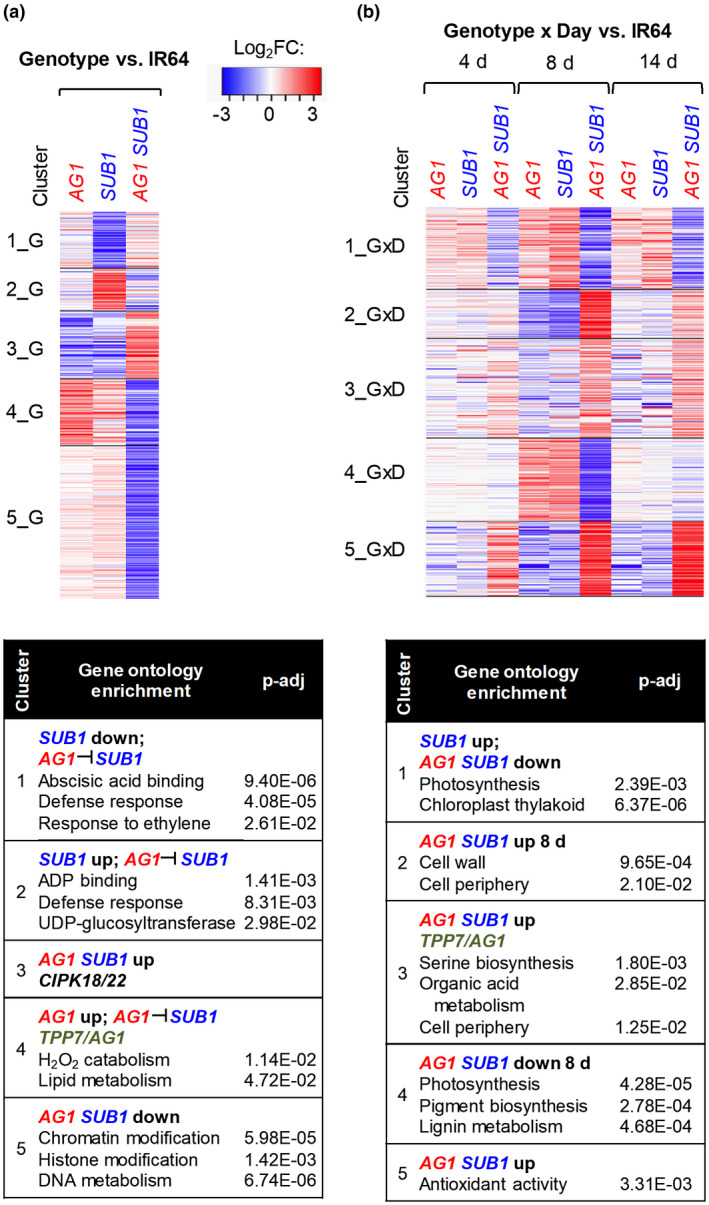
Genotypically controlled transcriptome changes during seedling establishment under prolonged submergence. (a) Clustering of DEGs identified in the genotypic comparisons (log_2_ FC > |1|; FDR < 0.05) of IR64(*AG1*), IR64(*SUB1*), and IR64(*AG1,SUB1*) with IR64 for all submergence days combined. (b) PAM clustering of DEGs identified by day comparison of IR64(*AG1*), IR64(*SUB1*), and IR64(*AG1,SUB1*) relative to the IR64 2 d transcriptome during specified submergence days (Genotype × Day vs. IR64; GxD). Representative GO enrichment of notable clusters and genes are listed. Genotype interactions diagrammed in Figure S6. A comprehensive list of DEGs, log_2_FC values, and GO term association are in Dataset S2

A multifactorial analysis of variance (ANOVA) was performed to resolve major genotypic signatures of the three introgression lines relative to IR64. To do so, the average log_2_CPM value across all sampling dates of IR64(*AG1*), IR64(*SUB1*), and IR64(*AG1,SUB1*) were compared with the average log_2_CPM value across all sampling days of IR64 for each transcript as depicted in Figure S6 (Genotype vs. IR64; Dataset S2a, b, c). This consolidated the data into three comparisons, identifying 817 gene transcripts that were differentially enriched or depleted relative to IR64 (log_2_FC [fold change] > |1|; FDR < 0.05) (Figure 4a). By sorting these into five clusters and using GO enrichment, we found that IR64(*SUB1*) had depleted levels of mRNAs associated with abscisic acid (ABA) and ethylene responses (cluster 1_G) and increased levels of ADP binding and F‐box proteins for proteolysis (cluster 2_G). In clusters 3_G and 4_G, the enriched mRNAs of IR64(*AG1*) and IR64(*SUB1*) were more similar to one another than the pyramided line, which had lower levels of these transcripts compared to IR64. The similarity between IR64(*AG1*) and IR64(*SUB1*) transcriptomes is consistent with the greater early shoot elongation (6 to 12 d) and better survival of prolonged submergence (Figure 2b,c). IR64(*AG1,SUB1*) was distinguished by elevated transcripts of calcium dependent kinases (cluster 3_G) and depleted transcripts associated with hydrogen peroxide catabolism (cluster 4_G) and chromatin structure/modification (cluster 5_G). Clusters 1_G and 2_G patterns indicate that *TPP7* acts epistatically to *SUB1A‐1* when pyramided in IR64. The other three clusters indicate that synergistic interactions arise when the two flooding loci are pyramided. Some genotypic differences observed in this and subsequent comparisons may reflect insertion/deletion and copy number and other chromosomal variations between genotypes, such as the known deletion encompassing *TPP7* in IR64.

A second multifactorial ANOVA was performed to evaluate genotype‐by‐day (GxD) interactions, identifying genes that were distinctly regulated in one or more genotypes at a specific time point relative to IR64 (Figure 4b; Dataset S2a, d, e). Gene log_2_CPM values at 2 d in IR64(*AG1*), IR64(*SUB1*), and IR64(*AG1,SUB1*) were individually compared to the 2 d log_2_CPM value of IR64 as illustrated in Figure S6. The resulting genotype versus IR64 values were individually compared to the log_2_CPM of 4, 8, and 12 d for each genotype. This identified 742 genes with specific regulation relative to IR64 (Figure 4b; Dataset S2a, d, e). Clustering of the GxD values that were differentially enriched or depleted in at least one genotype (log_2_ FC > |1|; FDR < 0.05) confirms the developmental transition between 4 and 8 d and provides a condensed view of the similarities between IR64(*AG1*) and IR64(*SUB1*) that contrast with IR64(*AG1,SUB1*). For example, cluster 1_GxD exposes the precocious activation of genes associated with photosynthetic enzyme complexes by *SUB1A‐1* at 8 d. Cluster 4_GxD genes are also enriched for photosynthesis, photorespiration, and light signaling, but these were similarly regulated in IR64(*AG1*) and IR64(*SUB1*) and delayed in IR64(*AG1,SUB1*). By contrast, clusters 2_GxD and 5_GxD group genes associated with cell wall biosynthesis and antioxidant metabolism that are amplified in the pyramided genotype at 8 d.

For further resolution, we performed pairwise comparisons between genotypes at each submergence time point (*i.e*., *AG1* vs. the other three genotypes) to identify DEGs and enriched gene ontologies as illustrated in Figures S6 and S7; Dataset S3d–k). Collectively, this systematic analysis determined that *AG1* and *SUB1A‐1* influence the timing of expression of genes associated with chloroplast biogenesis and amplitude of regulation of genes associated with nutrient mobilization, cell growth, immunity, and chromatin remodeling. *TPP7* and *SUB1A‐1* can act synergistically or antagonistically in the pyramided genotype. Counter to our initial hypothesis, *SUB1A‐1* influences the transcriptome during early seedling development, despite its low steady‐state transcript abundance.

### 3.7 Acceleration of underwater chloroplast development by *SUB1A‐1* is dampened by *TPP7*

A major finding is that IR64(*SUB1*) accelerates the activation of genes associated with the transition to photoautotrophy by day 4 of underwater development (Figures 3c and 4a,b; Figure S7). Clusters 7 and 8 of the time course analysis of the log_2_CPM data (Figure 3c) identify upregulated genes involved in chlorophyll biosynthesis. These include chlorophyll synthase (*GUN4*, LOC_Os11g16550) and protochlorophyllide reductases (*PORA*, LOC_Os04g58200; *PORB/FADED GREEN LEAF*, LOC_Os10g35370) as well as numerous chlorophyll a/b‐binding (*CAB*) proteins (i.e., a type I *CAB* already elevated in IR64(*SUB1*) at 2 d (LOC_Os01g52240)). The transcription factor *GOLDEN2‐LIKE 1* (*GLK1,* LOC_Os06g24070) is associated with chloroplast biogenesis (Nakamura et al., 2009). *GLK1* and *PORB* mRNAs were significantly higher in IR64(*AG1*) compared to IR64(*SUB1*) at day 2 (Figure S7a; cluster 1_A; Dataset S3f,g), but this trend is reversed by 4 d (Figure 3c, cluster 7; Figure S7b, cluster 8_S; Dataset S3h,i). A similar pattern was observed for *PORA*. The precociously upregulated genes included nucleus‐encoded photosystem I and II components, assembly factors, cytochrome *b6f,* ATP synthase subunits, and two plastid RNA polymerase sigma factors involved in transcription of plastid‐encoded photosystem machinery (*SIG2*, LOC_Os03g16430; *SIG6*, LOC_Os08g14450). Also advanced were mRNAs encoding light signaling components, ferredoxin, Calvin cycle (*i.e*., several Rubisco small subunit genes), and photorespiratory enzymes (*i.e., GLYCOLATE OXIDASE 1* [LOC_Os03g57220] and *3* [LOC_Os04g53210]). IR64(*AG1*) only slightly lagged behind IR64(*SUB1*) in the activation of chloroplast biogenesis and photosynthesis genes (Figure S7b, cluster 9_S); moreover, the delay was less in IR64(*AG1,SUB1*) than in IR64. Thus, introgression of *SUB1A‐1* or *TPP7* advances upregulation of genes associated with underwater chloroplast biogenesis in the IR64 background, but epistatic interaction between these genes delays the activation of genes associated with this critical transition in the pyramided line. Introgression of *SUB1A‐1* enables the *japonica* cultivar M202(*SUB1*) to recover photosystem II activity more quickly following prolonged darkness or vegetative‐stage submergence (Alpuerto et al., 2016; Fukao et al., 2011), although genes encoding the photosynthetic apparatus show only a modest advancement during recovery following short‐term submergence (Locke et al., 2017). The precocious activation of photoautotrophy during underwater development by *SUB1A‐1* may enable earlier production of photoassimilates and oxygen in the submerged seedlings and could account for the rapid increase in shoot length between day 4 and 8 in the IR64(*SUB1*) (Figure 2b). A role of *SUB1A‐1* in the transition to photoautotrophy has not been reported previously.

### 3.8 *SUB1A‐1* advances activation of genes associated with wax biosynthesis and deposition and control of ROS

Accompanying the upregulation of genes associated with photosynthetic competence was the elevation of genes involved in cuticular wax biosynthesis and deposition. These included *HYDROXYSTEROID DEHYDROGENASE 1*/*LEAF GAS FILM 1* (*HSD1*/*LGF1*; LOC_Os11g30560) and an ATP‐binding cassette transporter (*ABCG5/RCN1*; LOC_Os03g17350) (Figure 3c, clusters 7 and 8; Figure S7b, cluster 9_S). Mutant analysis demonstrated that *LGF1* is important in cuticle establishment and facilitates the formation of a thin gas film on the surface of submerged leaves (Kurokawa et al., 2018). *RCN1* is a plasma membrane transporter required for suberin deposition in the root hypodermis (endodermis) during waterlogging (Shiono et al., 2014). *RCN1* upregulation in shoot tissue may relate to cuticle estabilshment. Leaf gas film formation during submergence boosts gas exchange for photosynthesis (Colmer & Pedersen, 2008; Pedersen, Rich, & Colmer, 2009). Winkel, Pedersen, Ella, Ismail, and Colmer (2014) showed that underwater gas film retention, and net photosynthesis was similar between Swarna‐*SUB1* and Swarna. However, the *SUB1*‐donating FR13A displayed relatively higher gas film retention and photosynthesis, raising the possibility that early synthesis and deposition of a lipid barrier on the leaf epidermis may enhance submergence tolerance.

Photosynthesis, as well as other processes, produce ROS. In addition to their toxic effects, ROS are important for signaling, cell proliferation, and differentiation (Mittler, 2017). Consistent with the evidence of precocious chloroplast development, IR64(*SUB1*) had a higher abundance of transcripts associated with antioxidants at 4 d, compared to the other genotypes (Figure 3c, clusters 7 and 8). *SUB1A‐1* is known to limit the formation of ROS during submergence and upon desubmergence (Fukao et al., 2011), but the connection between these observations is not known.

### 3.9 Interactions between *TPP7* and *SUB1A‐1* are associated with starch catabolism, sucrose transport, anaerobic metabolism, and cell elongation

**FIGURE 5:**
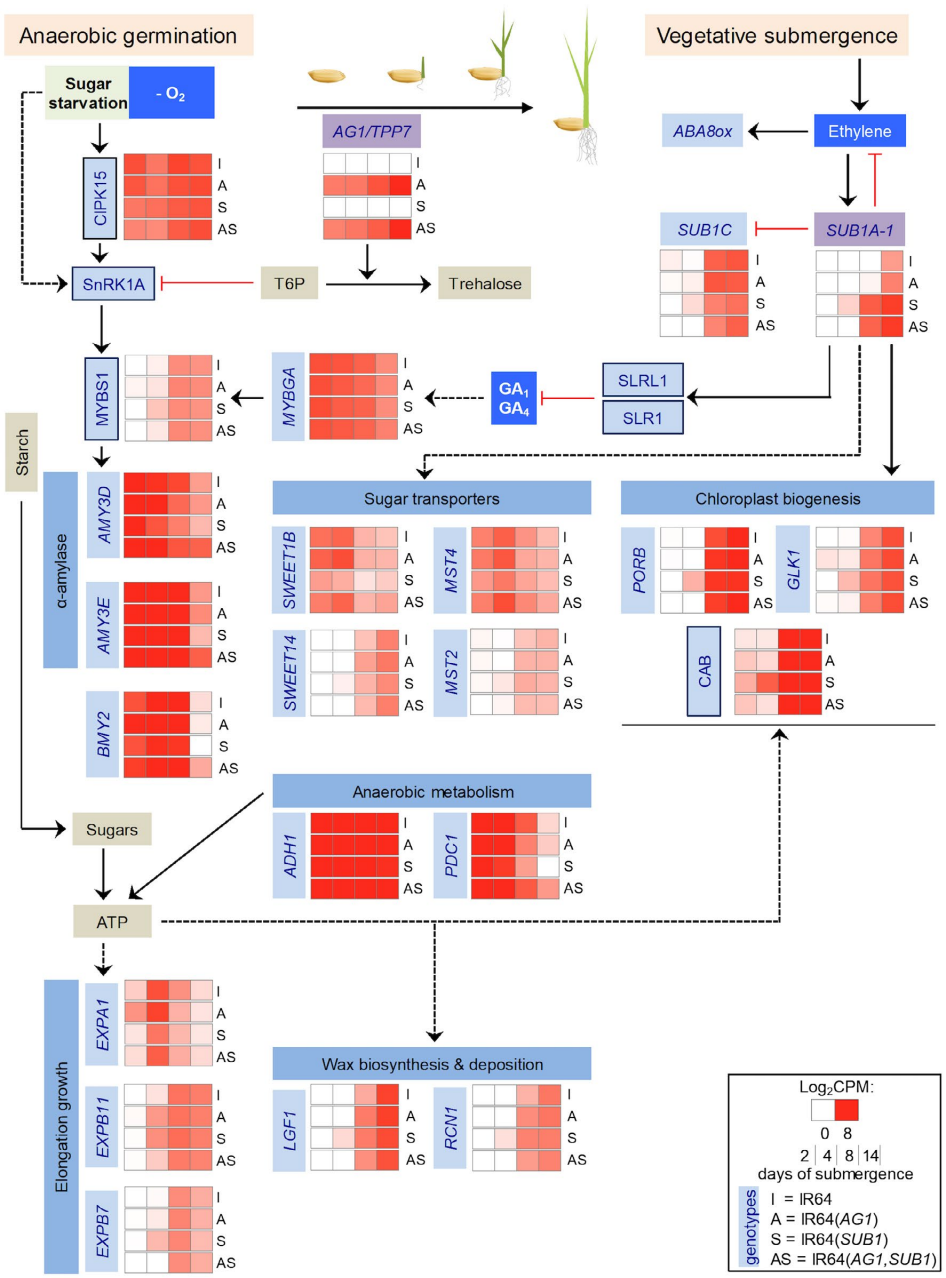
Overview of pathways involved in anaerobic germination and vegetative submergence influenced by *AG1*/*TPP7* and *SUB1*/*SUB1A‐1* respectively. The heatmap displays mean log_2_CPM values (representing on a scale of 0 to 8) of genes in embryo‐coleoptile‐shoot tissue. Proteins are displayed in black boxes, and genes are italicized. The left panel shows the mobilization of seed reserves that fuel elongation growth during anaerobic germination. The right panel shows the suppression of elongation growth during vegetative submergence. During anaerobic germination, low oxygen and sugar starvation promote the upregulation of *CALCINEURIN B‐LIKE INTERACTING PROTEIN KINASE 15* (*CIPK15*, LOC_Os11g02240) that promotes activation of the catalytic subunit of the energy‐sensing SUCROSE NON‐FERMENTING‐1‐RELATED PROTEIN KINASE 1 (SnRK1A, LOC_Os05g45420). SnRK1A activation enables the sugar starvation‐responsive MYBS1 (LOC_Os01g34060) transcription factor to enhance *α‐AMYLASE* transcription (*i.e., AMY3D*, LOC_Os08g36910; *AMY3E*, LOC_Os08g36900). During germination GA‐regulated MYB (*MYBGA*, LOC_Os01g59660) acts synergistically with MYBS1 to upregulate *AMYs*. These *AMYs* (and possibly *BMY2,* LOC_Os07g35940) hydrolyze endospermic starch into sugars that can be consumed in anaerobic ATP production within the embryo and developing seedling tissue. Two genes, *PYRUVATE DECARBOXYLASE 1* (*PDC1,* LOC_Os05g39310) and *ALCOHOL DEHYDROGENASE 1* (*ADH1,* LOC_Os11g10480) are vital during anaerobic respiration to produce ATP for growth and survival. This is accompanied by an elevation of *EXPANSINs* (*i.e*., *EXPA1*, LOC_Os04g15840; *EXPB11*, LOC_Os02g44108; *EXPB7*, LOC_Os03g01270, etc.). Trehalose‐6‐phosphate (T6P), a sensor of intracellular sucrose status, inhibits SnRK1A activation reducing catabolic metabolism. TPP7 (LOC_Os09g20390) catalyzes T6P conversion to trehalose, reducing the T6P to sucrose ratio to promote SnRK1A expression and coleoptile elongation. Submergence of vegetative‐stage plants entraps ethylene in shoot tissues, promoting the ethylene‐responsive transcription factor SUB1A‐1 to negatively regulate ethylene production. SUB1A‐1 stabilizes two gibberellin (GA) signaling repressors, GRAS domain transcription factors SLENDER RICE 1 (SLR1, LOC_Os03g49990) and SLR1‐LIKE 1 (SLRL1, LOC_Os01g45860) that reduce GA‐responsiveness. Our data indicate that SUB1A‐1 may also influence the upregulation of *SUGARS WILL EVENTUALLY BE EXPORTED TRANSPORTERs* (*i.e., SWEET1B,* LOC_Os05g35140; *SWEET14,* LOC_Os11g31190) sugar transporters (*i.e., MST2,* LOC_Os03g39710; *MST4,* LOC_Os03g11900) gene family members. Our data further indicate that in seedlings developing underwater SUB1A‐1 directly or indirectly promotes early activation of genes associated with chloroplast development (*i.e.,* type I chlorophyll a/b‐binding protein *CAB,* LOC_Os01g52240), enzymes involved in chlorophyll biosynthesis (*i.e., PORB*, LOC_Os10g35370) and a transcription factor associated with chloroplast biogenesis (*GOLDEN2‐LIKE 1*, *GLK1,* LOC_Os06g24070). Genes involved in leaf cuticular wax biosynthesis, including hydroxysteroid dehydrogenase (*LGF1*, LOC_Os11g30560) and deposition (ATP binding cassette transporter *RCN1*, LOC_Os03g17350), are also upregulated early by *SUB1A‐1*

Based on the rise in *SUB1A‐1* transcript accumulation over the submergence time course, we hypothesized that the pyramided genotype would limit mRNAs associated with endosperm or shoot nutrient catabolism or mobilization. To evaluate this, we compared the log_2_CPM values of transcripts encoding known players in the hydrolysis of seed reserves and elongation growth during anaerobic germination, leveraging the statistical outcome of the genotypic comparisons (Dataset S1d; Dataset S3d‐k). This analysis is distilled in Figure 5.

Considering the activation of starch catabolism during anaerobic germination, IR64(*SUB1*) and IR64(*AG1,SUB1*) had lower *CIPK15* (LOC_Os11g02240) transcripts than IR64(*AG1*) and IR64 at 2 d, whereas levels of mRNA encoding its target SnRK1A (LOC_Os05g45420) were indistinguishable among genotypes at all time points (Dataset S1d). Shoots of vegetative stage (21 d post‐germination) M202(*SUB1*) seedlings maintain higher T6P content and significantly dampen *CIPK15* mRNA with no distinction in *SnRK1A* mRNA levels when submerged, as compared to near‐isogenic M202 (Locke et al., 2017). Here we found that the upregulation of *MYBS1* (LOC_Os01g34060) in IR64(*AG1*) precedes that of IR64(*SUB1*). *MYBGA* (LOC_Os01g59660)*,* which acts synergistically with MYBS1 during anaerobic germination (Hong et al., 2012)*,* was upregulated similarly in all genotypes, indicating that *SUB1A‐1*’s underwater dampening of GA‐responsiveness in shoot tissue is inconsequential to the upregulation of *MYBGA*. The starch catabolism genes *AMY3D* (LOC_Os08g36910)*, AMY3E* (LOC_Os08g36900), and *β‐AMYLASE 2* (*BMY2*; LOC_Os07g35940) were high in all genotypes at 2 to 8 d but tapered off the most rapidly in IR64(*SUB1*) and the most slowly in the pyramided genotype. *AMY3D* transcripts declined as early as by 8 d in IR64(*SUB1*) and IR64(*AG1*). These three *AMY* genes were significantly upregulated in IR64(*AG1,SUB1*) compared to other genotypes at 14 d (Figure S7c, cluster 6_AS, Dataset S3j,k), suggesting that it is less able to temper underwater catabolic metabolism.

Next, we investigated genes encoding transporters involved in the regulation of resource allocation (*i.e*., from the endosperm (source) to the elongating coleoptile and developing plumule (sink) or from photosyntheticl to non‐photosynthetic cells). Based on studies in multiple plant species, carbohydrate allocation to sink tissues is associated with elevated *TPP* gene transcripts and T6P low content (Paul, Gonzalez‐Uriarte, Griffiths, & Hassani‐Pak, 2018). In maize (*Zea mays* L.), expression of rice *TPP1* in the phloem cells of the ear pith and florets promotes transcription of genes encoding SUGARS WILL EVENTUALLY BE EXPORTED TRANSPORTERs (SWEETs), enhancing sucrose transport from leaves to developing kernels (Nuccio et al., 2015; Oszvald et al., 2018). Several *SWEET*s, *SUCROSE TRANSPORTERS* (*SUTs*), and *MONOSACCHARIDE TRANSPORTERS* (*MSTs*) genes showed temporal and genotypic regulation (Figure 3c; Dataset S1d). *SWEET1B* (LOC_Os05g35140, cluster_2) and *MST4* (LOC_Os03g11900, cluster_2) mRNAs were abundant in all genotypes at 2 and 4 d, but at significantly lower levels in IR64(*SUB1*) at 2 d (Figure 5). By contrast, *SWEET14* (LOC_Os11g31190, cluster_7) and *MST2* (LOC_Os03g39710*,* cluster_8) mRNAs were elevated at the later time points, with slightly early upregulation in IR64(*SUB1*). The regulation of these genes in the pyramided genotype was similar to that of IR64(*AG1*). Plausibly, the predicted monosaccharide transporter SWEET1B participates in the movement of glucose or maltose, generated by endospermic starch hydrolysis, to the elongating coleoptile and developing plumule, whereas the mobilization of photoassimilates by phloem parenchymal SWEET14 (Chandran, 2015; Chen, 2013) could be needed to maintain cell activity during submergence.

Maintenance of ATP production under hypoxic conditions requires not only sugars but the regeneration of NAD^+^ through anaerobic fermentation (Bailey‐Serres & Voesenek, 2008). Key enzymes for this process include *PYRUVATE DECARBOXYLASE 1* (*PDC1*, LOC_Os05g39310; cluster 6_AS), *ADH1* and *ADH2* (LOC_Os11g10480; LOC_Os11g10510; Figure S7c, cluster 7_AS). The pyramided line continued to upregulate genes associated with anaerobic metabolism and alcoholic fermentation at 8 and 14 d relative to the other genotypes (Figure 5; Dataset S1d and Dataset S3j, k). Although *SUB1A‐1* dampens starch catabolism during submergence, it promotes the upregulation of *ADH* and *PDC* genes and enzymatic activities (Fukao et al., 2006). Our observations indicate that IR64(*AG1,SUB1*) may differ from both IR64(*AG1*) and IR64(*SUB1*) in the regulation of anaerobic metabolism and maintenance of energy homeostasis.

The use of ATP for growth and anisotropic cell expansion may also differ between genotypes. Corroborating early *AG1*‐promoted elongation growth (Figure 2b), IR64(*AG1*) had high expression of *EXPA1* (LOC_Os04g15840; 4–8 fold at 2 d), relative to the other genotypes (Figure 5; Figure S7a, cluster 1_A). Between 4 to 8 d, IR64(*SUB1*) had the second‐highest elongation growth after IR64(*AG1*) (Figure 2b) and greater expression of *EXP*s and other cell wall‐related genes (cluster 3_S and 9_S), including *EXPB11* (LOC_Os02g44108) and *EXPB7* (LOC_Os03g01270). The early and sustained dampening of elongation in the pyramided line is consistent with the significant early downregulation of *EXPA6* (LOC_Os03g21820), expressed highly at the base of coleoptile in anaerobic seedlings (Narsai et al., 2015) (Figure S7c, cluster 8_AS). Corroborating the *AG1*‐induced growth, IR64(*AG1*) had more cell wall synthesis‐associated transcripts compared to IR64(*SUB1*) and earlier induction of cell development compared to IR64(*AG1,SUB1*) at 8 and 14 d (Figure S7a; cluster 5_A). By 8 d, IR64(*SUB1*) dampened genes associated with cell division, wall/cuticle development, and fatty acid biosynthesis (Figure S7a,b, cluster 5_A, cluster 4_S). In IR64(*AG1,SUB1*), cell division, cell wall synthesis, and elongation growth were among the transcripts that were suppressed at 2 d (Figure S7c, cluster 8_AS) but remained high at 14 d (cluster 6_AS). This differential transcript regulation is consistent with the observed underwater development of the four genotypes.

### 3.10 DNA repair, cell cycle, and epigenetic regulation during early germination

DNA damage repair, DNA synthesis, and activation of mitosis are critical to germination‐performance and seedling (Waterworth, Bray, & West, 2019). We identified differential regulation of genes associated with these processes in the four genotypes. Replication protein A (RPA), a heterotrimeric DNA binding protein, is necessary for DNA replication, repair, and recombination (Aklilu & Culligan, 2016). The transcripts of *RPA70B* (LOC_Os03g11540) and *RPA32* (LOC_Os02g58220) are expressed strongly in proliferating tissues of rice (Ishibashi et al., 2001). Levels of *RPA70B* mRNA were significantly elevated by 2 d in IR64(*AG1*) and by 4 d in IR64(*SUB1*) along with *RPA32* (cluster 7; Dataset S1d). Formation of a pre‐replication complex precedes entry into the S‐phase of the cell cycle and requires the sequential association of origin recognition complex (ORC), DNA replication factor CDT1, CELL DIVISION CONTROL 6 (CDC6), and MINI‐CHROMOSOME MAINTENANCE 2–7 (MCM2‐7) (Bryant & Aves, 2011). IR64(*SUB1*) precociously upregulated transcripts encoding several of these proteins at day 2 or 4 of underwater development, including *ORC1* (LOC_Os06g08790), *CDT1A* (LOC_Os04g10650, cluster 8), *CDT1B* (LOC_Os10g34820, cluster 4), and *CDC6* (LOC_Os01g63710; Dataset S1d). Rice *MCM2* (LOC_Os11g29380, cluster 7) is generally expressed but downregulated by a nutrient shortage, reflecting the need for adequate energy supply for cell cycle progression (Cho, Kim, Kim, & Choi, 2008). *MCM2* and *MMC7* (LOC_Os12g37400, cluster 8) were significantly downregulated in IR64(*AG1,SUB1*) by comparison to IR64(*AG1*) and IR64(*SUB1*) at 2 d. IR64(*SUB1*) also significantly elevated levels of mRNAs encoding two B‐type cyclin‐dependent kinases (*CDKB2;1*, LOC_Os08g40170, *CYCB2;2*, LOC_Os06g51110) and numerous cyclins after 4 d of germination underwater (Dataset S1d).

Epigenetic regulation, including DNA methylation and histone modifications, is indispensable for plant development and environmental response. IR64(*SUB1*) had elevated levels of mRNAs encoding *DNA METHYLTRANSFERASE 1B* (LOC_Os07g08500) associated with CG methylation and *CHROMOMETHYLASE 3A* (LOC_Os10g01570) associated with CHG methylation and transposon silencing (Dataset S1d). We saw little difference in expression of mRNAs encoding histone‐modifying enzymes, but several core histone protein genes were upregulated in IR64(*SUB1*). The latter contrasts with the observation by Locke et al. (2017) that M202(*SUB1*) shoots limit upregulation of histone genes in response to submergence. Overall, our data suggest that *SUB1A‐1* promotes cell cycle activity earlier during underwater germination, and epistatic interaction between *SUB1A‐1* and *TPP7* may impact cell division as well as epigenetic modifications.

## 4 CONCLUSIONS

This study demonstrates that seeds encoding the flooding trait loci *AG1/TPP7* and *SUB1/SUB1A‐1,* in the widely grown IR64 genetic background, can be used for direct‐seeding into paddies of controlled depth and will display submergence tolerance if inundated after establishment of vegetative‐stage seedlings. The direct‐seeding advantage provided by *TPP7* involves early and efficient mobilization of seed reserves to fuel coleoptile elongation (Kretzschmar et al., 2015). This germination and seedling establishment trait is not negatively impacted by the addition of *SUB1A‐1* to the genetic background (Septiningsih et al., 2009), despite *SUB1A‐1*’s opposing function to control carbohydrate catabolism and elongation growth during a deep submergence event (Fukao et al., 2006; Yeung et al., 2019). Yet, growth, survival, and transcriptomic data demonstrate that *TPP7* and *SUB1A‐1* interact when seeds are sown underwater, and the developing seedlings restrained from submergence escape through the transition to photoautotrophy.

In the pyramided line, *TPP7* and *SUB1A‐1* act epistatically in a temporal and likely spatial (tissue‐ and cell‐specific) manner by dampening the activation of genes associated with cell division, epigenetic modifications, photoautotrophy, and by delaying and prolonging the expression of genes associated with carbohydrate catabolism and elongation growth. All of these factors may contribute to lower germination rates, poor seedling establishment, and ultimately reduced survival after desubmergence. Our data indicate that the pyramided genotype utilizes endospermic and photoassimilate starch in a temporally distinct manner. As compared to IR64(*AG1*) and IR64(*SUB1*), this may slow the initiation of seed starch hydrolysis but prolong its catabolism. Although the metabolite data on whole tissues were largely uninformative, the transcriptome data raise the hypothesis that the pyramided genotype is programmed by *TPP7* to tap endosperm starch efficiently, but that this is dampened at the level of carbohydrate transport by *SUB1A‐1* (*i.e., SWEET1B*). We discovered that *SUB1A‐1* confers early investment of energy into the conversion to photoautotrophy, but this may be limited in the pyramided genotype by the maintenance of low T6P levels that promote seed nutrient use. Our findings indicate that underwater seedling establishment includes the switch from reliance on seed reserves for coleoptile elongation to the biogenesis of chloroplasts in leaflets to provide photosynthate to advance development. Further understanding of this balance will require monitoring of T6P dynamics, metabolite flux, and gene activity in specific tissues or individual cells of submerged germinating rice.

The *AG1* and *SUB1* combination may be beneficial in many rice plantations, but genetic interactions between these high‐value loci could be disadvantageous if seedlings fail to escape after the transition to vegetative growth. Genotypes with *SUB1A‐1* can also be less productive when plants are forced to endure prolonged stagnant flooding during later vegetative development (Kato, Collard, Septiningsih, & Ismail, 2019). Nonetheless, there are genetic combinations that include *SUB1* that display stagnant flooding tolerance and other anaerobic germination loci that may be effective in combination with *SUB1* (Toledo et al., 2015). Knowledge from this study can help rice researchers to understand the consequence of pyramiding *AG1*, *SUB1,* and other valued loci in farmers’ preferred varieties.

## CONFLICT OF INTEREST

The authors declare no conflict of interest associated with the work described in this manuscript.

## AUTHOR CONTRIBUTIONS

R.A., E.S., and J.B.‐S. conceived the project; R.A., A.M.I., E.S., and J.B.‐S. designed the experiments; R.A. performed the stress experiments and acquired samples and data at the International Rice Research Institute and the University of California, Riverside; A.M.I., E.S., and J.B.‐S. supervised the experiments; R.A. acquired and analyzed agronomic, physiological, and biochemical data; R.A. constructed RNA‐sequencing libraries; M.H. performed the bioinformatic analyses; R.A., M.H., E.Y., and J.B.‐S. analyzed the transcriptomic data and constructed figures; E.Y. performed critical data interpretation and curation; J.C.I.I. and M.D.B. developed IR64(*AG1,SUB1*) and prepared seed materials; R.A., M.H., A.L., and Z.J. designed and implemented the statistical methodology; R.A. wrote the first draft of the manuscript; E.Y. and J.B.‐S. recrafted the manuscript with assistance of R.A. and M.H.; A.L., A.M.I., and E.S. reviewed the manuscript that was approved by all other authors; R.A. acquired fellowship funding with sponsorship of E.S. and J.B.‐S.; E.S., A.M.I., and J.B.‐S. acquired research funding. J.B‐S. agrees to serve as the author responsible for contact and ensures communication.

## Supporting information

Fig S1

Fig S2

Fig S3

Fig S4

Fig S5

Fig S6

Fig S7a–b

Fig S7C_D

Table S1

Dataset S1

Dataset S2

Dataset S3

Fig S1‐S7

Supplementary Material

## References

[pld3240-bib-0001] Aklilu, B. B. , & Culligan, K. M. (2016). Molecular evolution and functional diversification of Replication Protein A1 in plants. Frontiers in Plant Science, 7, 33. 26858742 10.3389/fpls.2016.00033 PMC4731521

[pld3240-bib-0002] Alpuerto, J. B. , Hussain, R. M. F. , & Fukao, T. (2016). The key regulator of submergence tolerance, *SUB1A*, promotes photosynthetic and metabolic recovery from submergence damage in rice leaves. Plant, Cell and Environment, 39, 672–684. 10.1111/pce.12661 26477688

[pld3240-bib-0003] Angaji, S. A. , Septiningsih, E. M. , Mackill, D. J. , & Ismail, A. M. (2009). QTLs associated with tolerance of flooding during germination in rice (*Oryza sativa* L.). Euphytica, 172, 159–168. 10.1007/s10681-009-0014-5

[pld3240-bib-0004] Backman, T. W. H. , & Girke, T. (2016). systemPipeR: NGS workflow and report generation environment. BMC Bioinformatics, 17, 388. 27650223 10.1186/s12859-016-1241-0 PMC5029110

[pld3240-bib-0005] Bailey‐Serres, J. , Fukao, T. , Ronald, P. , Ismail, A. , Heuer, S. , & Mackill, D. (2010). Submergence tolerant rice: *SUB1*’s journey from landrace to modern cultivar. Rice, 3, 138–147. 10.1007/s12284-010-9048-5

[pld3240-bib-0006] Bailey‐Serres, J. , & Voesenek, L. A. C. J. (2008). Flooding stress: Acclimations and genetic diversity. Annual Review of Plant Biology, 59, 313–339. 10.1146/annurev.arplant.59.032607.092752 18444902

[pld3240-bib-0007] Bryant, J. A. , & Aves, S. J. (2011). Initiation of DNA replication: Functional and evolutionary aspects. Annals of Botany, 107, 1119–1126. 10.1093/aob/mcr075 21508040 PMC3091809

[pld3240-bib-0008] Chamara, B. S. , Marambe, B. , Kumar, V. , Ismail, A. M. , Septiningsih, E. M. , & Chauhan, B. S. (2018). Optimizing sowing and flooding depth for anaerobic germination‐tolerant genotypes to enhance crop establishment, early growth, and weed management in dry‐seeded rice (*Oryza sativa* L.). Frontiers in Plant Science, 9, 1654 10.3389/fpls.2018.01654 30532759 PMC6265439

[pld3240-bib-0009] Chandran, D. (2015). Co‐option of developmentally regulated plant SWEET transporters for pathogen nutrition and abiotic stress tolerance. IUBMB Life, 67, 461–471. 26179993 10.1002/iub.1394

[pld3240-bib-0010] Chen, L. Q. (2013). SWEET sugar transporters for phloem transport and pathogen nutrition. New Phytologist, 201, 1150–1155. 10.1111/nph.12445 24649486

[pld3240-bib-0011] Chen, Y. S. , Ho, T. H. D. , Liu, L. , Lee, D. H. , Lee, C. H. , Chen, Y. R. , … Yu, S. M. (2019). Sugar starvation‐regulated MYBS2 and 14–3‐3 protein interactions enhance plant growth, stress tolerance, and grain weight in rice. Proceedings of the National Academy of Sciences of the United States of America, 116(43), 21925–21935. 10.1073/pnas.1904818116 31594849 PMC6815185

[pld3240-bib-0012] Cho, J. H. , Kim, H. B. , Kim, H.‐S. , & Choi, S.‐B. (2008). Identification and characterization of a rice MCM2 homologue required for DNA replication. BMB Reports, 41, 581–586. 18755073 10.5483/bmbrep.2008.41.8.581

[pld3240-bib-0013] Colmer, T. D. , & Pedersen, O. (2008). Underwater photosynthesis and respiration in leaves of submerged wetland plants: Gas films improve CO_2_ and O_2_ exchange. New Phytologist, 177, 918–926. 18086222 10.1111/j.1469-8137.2007.02318.x

[pld3240-bib-0014] Das, K. K. , Panda, D. , Sarkar, R. K. , Reddy, J. N. , & Ismail, A. M. (2009). Submergence tolerance in relation to variable floodwater conditions in rice. Environmental and Experimental Botany, 66, 425–434. 10.1016/j.envexpbot.2009.02.015

[pld3240-bib-0015] Ella, E. S. , & Setter, T. L. (1999). Importance of seed carbohydrates in rice seedling establishment under anoxia. Acta Horticulturae, 504, 209–218.

[pld3240-bib-0016] Figueroa, C. M. , & Lunn, J. E. (2016). A tale of two sugars: Trehalose 6‐phosphate and sucrose. Plant Physiology, 172, 7–27. 10.1104/pp.16.00417 27482078 PMC5074632

[pld3240-bib-0017] Fukao, T. , & Bailey‐Serres, J. (2008). Submergence tolerance conferred by *Sub1A* is mediated by SLR1 and SLRL1 restriction of gibberellin responses in rice. Proceedings of the National Academy of Sciences of the United States of America, 105, 16814–16819. 10.1073/pnas.0807821105 18936491 PMC2575502

[pld3240-bib-0018] Fukao, T. , Xu, K. , Ronald, P. C. , & Bailey‐Serres, J. (2006). A variable cluster of ethylene response factor‐like genes regulates metabolic and developmental acclimation responses to submergence in rice. The Plant Cell, 18, 2021–2034. 10.1105/tpc.106.043000 16816135 PMC1533987

[pld3240-bib-0019] Fukao, T. , Yeung, E. , & Bailey‐Serres, J. (2011). The submergence tolerance regulator *SUB1A* mediates crosstalk between submergence and drought tolerance in rice. The Plant Cell, 23, 412–427. 10.1105/tpc.110.080325 21239643 PMC3051255

[pld3240-bib-0020] Gonzaga, Z. J. C. , Carandang, J. , Sanchez, D. L. , Mackill, D. J. , & Septiningsih, E. M. (2016). Mapping additional QTLs from FR13A to increase submergence tolerance in rice beyond *SUB1* . Euphytica, 209, 627–636. 10.1007/s10681-016-1636-z

[pld3240-bib-0021] Greenway, H. , & Setter, T. L. (1996). Is there anaerobic metabolism in submerged rice plants? A view point In Singh V. P., Singh R. K., Singh B. B., & Zeigler R. S. (Eds.), Physiology of stress tolerance in plants (pp. 11–30). Los Banos, Philippines: International Rice Research Institute.

[pld3240-bib-0022] Hirabayashi, Y. , Mahendran, R. , Koirala, S. , Konoshima, L. , Yamazaki, D. , Watanabe, S. , … Kanae, S. (2013). Global flood risk under climate change. Nature Climate Change, 3, 816–821. 10.1038/nclimate1911

[pld3240-bib-0023] Hong, Y. F. , Ho, T. H. D. , Wu, C. F. , Ho, S. L. , Yeh, R. H. , Lu, C. A. , … Yu, S. M. (2012). Convergent starvation signals and hormone crosstalk in regulating nutrient mobilization upon germination in cereals. The Plant Cell, 24, 2857–2873. 10.1105/tpc.112.097741 22773748 PMC3426119

[pld3240-bib-0024] Iftekharuddaula, K. M. , Ghosal, S. , Gonzaga, Z. J. , Amin, A. , Barman, H. N. , Yasmeen, R. , … Septiningsih, E. M. (2016). Allelic diversity of newly characterized submergence‐tolerant rice (*Oryza sativa* L.) germplasm from Bangladesh. Genetic Resources and Crop Evolution, 63, 859–867. 10.1007/s10722-015-0289-4

[pld3240-bib-0025] Iftekharuddaula, K. M. , Newaz, M. A. , Salam, M. A. , Ahmed, H. U. , Mahbub, M. A. A. , Septiningsih, E. M. , … Mackill, D. J. (2011). Rapid and high‐precision marker assisted backcrossing to introgress the SUB1 QTL into BR11, the rainfed lowland rice mega variety of Bangladesh. Euphytica, 178, 83–97. 10.1007/s10681-010-0272-2

[pld3240-bib-0026] Ishibashi, T. , Kimura, S. , Furukawa, T. , Hatanaka, M. , Hashimoto, J. , & Sakaguchi, K. (2001). Two types of replication protein A 70 kDa subunit in rice, *Oryza sativa*: Molecular cloning, characterization, and cellular & tissue distribution. Gene, 272, 335–343. 11470540 10.1016/s0378-1119(01)00555-8

[pld3240-bib-0027] Ismail, A. M. , Ella, E. S. , Vergara, G. V. , & Mackill, D. J. (2009). Mechanisms associated with tolerance to flooding during germination and early seedling growth in rice (*Oryza sativa*). Annals of Botany, 103, 197–209. 10.1093/aob/mcn211 19001425 PMC2707318

[pld3240-bib-0028] Ismail, A. M. , Johnson, D. E. , Ella, E. S. , Vergara, G. V. , & Baltazar, A. M. (2012) Adaptation to flooding during emergence and seedling growth in rice and weeds, and implications for crop establishment. AoB Plants, 2012: pls019. 22957137 10.1093/aobpla/pls019 PMC3434364

[pld3240-bib-0029] Ismail, A. M. , Singh, U. S. , Singh, S. , Dar, M. H. , & Mackill, D. J. (2013). The contribution of submergence‐tolerant (Sub1) rice varieties to food security in flood‐prone rainfed lowland areas in Asia. Field Crops Research, 152, 83–93. 10.1016/j.fcr.2013.01.007

[pld3240-bib-0030] Jung, K. H. , Seo, Y. S. , Walia, H. , Cao, P. , Fukao, T. , Canlas, P. E. , … Ronald, P. C. (2010). The submergence tolerance regulator *Sub1A* mediates stress‐responsive expression of *AP2/ERF* transcription factors. Plant Physiology, 152, 1674–1692. 20107022 10.1104/pp.109.152157 PMC2832257

[pld3240-bib-0031] Juntawong, P. , Girke, T. , Bazin, J. , & Bailey‐Serres, J. (2014). Translational dynamics revealed by genome‐wide profiling of ribosome footprints in *Arabidopsis* . Proceedings of the National Academy of Sciences, 111, E203–E212. 10.1073/pnas.1317811111 PMC3890782 24367078

[pld3240-bib-0032] Kato, Y. , Collard, B. C. Y. , Septiningsih, E. M. , & Ismail, A. M. (2019). Increasing flooding tolerance in rice: Combining tolerance of submergence and of stagnant flooding. Annals of Botany, 124, 1199–1210. 10.1093/aob/mcz118 PMC6944782 31306479

[pld3240-bib-0033] Kretzschmar, T. , Pelayo, M. A. F. , Trijatmiko, K. R. , Gabunada, L. F. M. , Alam, R. , Jimenez, R. , … Septiningsih, E. M. (2015). A trehalose‐6‐phosphate phosphatase enhances anaerobic germination tolerance in rice. Nature Plants, 1, 15124 10.1038/nplants.2015.124 27250677

[pld3240-bib-0034] Kurokawa, Y. , Nagai, K. , Huan, P. D. , Shimazaki, K. , Qu, H. , Mori, Y. , … Ashikari, M. (2018). Rice leaf hydrophobicity and gas films are conferred by a wax synthesis gene (LGF1) and contribute to flood tolerance. New Phytologist, 218, 1558–1569. 29498045 10.1111/nph.15070

[pld3240-bib-0035] Lasanthi‐Kudahettige, R. , Magneschi, L. , Loreti, E. , Gonzali, S. , Licausi, F. , Novi, G. , … Perata, P. (2007). Transcript profiling of the anoxic rice coleoptile. Plant Physiology, 144, 218–231. 10.1104/pp.106.093997 17369434 PMC1913783

[pld3240-bib-0036] Lee, K. W. , Chen, P. W. , Lu, C. A. , Chen, S. , Ho, T. H. D. , & Yu, S. M. (2009). Coordinated responses to oxygen and sugar deficiency allow rice seedlings to tolerate flooding. Science Signalling, 2, ra61 10.1126/scisignal.2000333 19809091

[pld3240-bib-0037] Lee, K. W. , Chen, P. W. , & Yu, S. M. (2014). Metabolic adaptation to sugar/O_2_ deficiency for anaerobic germination and seedling growth in rice. Plant, Cell and Environment, 37, 2234–2244. 10.1111/pce.12311 24575721

[pld3240-bib-0038] Li, Z. , & Trick, H. N. (2005). Rapid method for high‐quality RNA isolation from seed endosperm containing high levels of starch. BioTechniques, 38(6), 872–876. 16018547 10.2144/05386BM05

[pld3240-bib-0039] Lin, C.‐C. , Chao, Y.‐T. , Chen, W.‐C. , Ho, H.‐Y. , Chou, M.‐Y. , Li, Y.‐R. , … Shih, M.‐C. (2019). Regulatory cascade involving transcriptional and N‐end rule pathways in rice under submergence. Proceedings of the National Academy of Sciences of the United States of America, 116, 3300–3309. 10.1073/pnas.1818507116 30723146 PMC6386710

[pld3240-bib-0040] Locke, A. M. , Barding, G. A., Jr. , Sathnur, S. , Larive, C. K. , & Bailey‐Serres, J. (2017). Rice *SUB1A* constrains remodelling of the transcriptome and metabolome during submergence to facilitate post‐submergence recovery. Plant, Cell and Environment, 41(4), 721–736. 10.1111/pce.13094 29094353

[pld3240-bib-0041] Mackill, D. J. , Ismail, A. M. , Singh, U. S. , Labios, R. V. , & Paris, T. R. (2012). Chapter six ‐ Development and rapid adoption of submergence‐tolerant (Sub1) rice varieties In Sparks D. L. (Ed.), Advances in agronomy (pp. 299–352). Cham, Switzerland: Academic Press.

[pld3240-bib-0042] Magneschi, L. , Kudahettige, R. L. , Alpi, A. , & Perata, P. (2009). Comparative analysis of anoxic coleoptile elongation in rice varieties: Relationship between coleoptile length and carbohydrate levels, fermentative metabolism and anaerobic gene expression. Plant Biology, 11, 561–573. 10.1111/j.1438-8677.2008.00150.x 19538394

[pld3240-bib-0043] Mittler, R. (2017). ROS are good. Trends in Plant Science, 22, 11–19. 10.1016/j.tplants.2016.08.002 27666517

[pld3240-bib-0044] Mustroph, A. , Lee, S. C. , Oosumi, T. , Zanetti, M. E. , Yang, H. , Ma, K. , … Bailey‐Serres, J. (2010). Cross‐kingdom comparison of transcriptomic adjustments to low‐oxygen stress highlights conserved and plant‐specific responses. Plant Physiology, 152, 1484–1500. 10.1104/pp.109.151845 20097791 PMC2832244

[pld3240-bib-0045] Nakamura, H. , Muramatsu, M. , Hakata, M. , Ueno, O. , Nagamura, Y. , Hirochika, H. , … Ichikawa, H. (2009). Ectopic overexpression of the transcription factor OsGLK1 induces chloroplast development in non‐green rice cells. Plant and Cell Physiology, 50, 1933–1949. 10.1093/pcp/pcp138 19808806 PMC2775961

[pld3240-bib-0046] Narsai, R. , Edwards, J. M. , Roberts, T. H. , Whelan, J. , Joss, G. H. , & Atwell, B. J. (2015). Mechanisms of growth and patterns of gene expression in oxygen‐deprived rice coleoptiles. The Plant Journal, 82, 25–40. 10.1111/tpj.12786 25650041

[pld3240-bib-0047] Nghi, K. N. , Tondelli, A. , Valè, G. , Tagliani, A. , Marè, C. , Perata, P. , & Pucciariello, C. (2019). Dissection of coleoptile elongation in japonica rice under submergence through integrated genome‐wide association mapping and transcriptional analyses. Plant, Cell and Environment, 42, 1832–1846. 10.1111/pce.13540 30802973

[pld3240-bib-0048] Nuccio, M. L. , Wu, J. , Mowers, R. , Zhou, H.‐P. , Meghji, M. , Primavesi, L. F. , … Lagrimini, L. M. (2015). Expression of trehalose‐6‐phosphate phosphatase in maize ears improves yield in well‐watered and drought conditions. Nature Biotechnology, 33, 862–869. 10.1038/nbt.3277 26473199

[pld3240-bib-0049] Oszvald, M. , Primavesi, L. F. , Griffiths, C. A. , Cohn, J. , Basu, S. S. , Nuccio, M. L. , & Paul, M. J. (2018). Trehalose 6‐phosphate regulates photosynthesis and assimilate partitioning in reproductive tissue. Plant Physiology, 176, 2623–2638. 10.1104/pp.17.01673 29437777 PMC5884609

[pld3240-bib-0050] Paul, M. J. , Gonzalez‐Uriarte, A. , Griffiths, C. A. , & Hassani‐Pak, K. (2018). The role of trehalose 6‐phosphate in crop yield and resilience. Plant Physiology, 177, 12–23. 10.1104/pp.17.01634 29592862 PMC5933140

[pld3240-bib-0051] Pedersen, O. , Rich, S. M. , & Colmer, T. D. (2009). Surviving floods: Leaf gas films improve O_2_ and CO_2_ exchange, root aeration, and growth of completely submerged rice. The Plant Journal, 58, 147–156. 10.1111/j.1365-313X.2008.03769.x 19077169

[pld3240-bib-0052] Ramon, M. , Dang, T. V. T. , Broeckx, T. , Hulsmans, S. , Crepin, N. , Sheen, J. , & Rolland, F. (2019). Default activation and nuclear translocation of the plant cellular energy sensor SnRK1 regulate metabolic stress responses and development. The Plant Cell, 31, 1614–1632. 10.1105/tpc.18.00500 31123051 PMC6635846

[pld3240-bib-0053] Schmitz, A. J. , Folsom, J. J. , Jikamaru, Y. , Ronald, P. , & Walia, H. (2013). SUB1A‐mediated submergence tolerance response in rice involves differential regulation of the brassinosteroid pathway. New Phytologist, 198, 1060–1070. 23496140 10.1111/nph.12202

[pld3240-bib-0054] Septiningsih, E. M. , Collard, B. C. Y. , Heuer, S. , Bailey‐Serres, J. , Ismail, A. M. , & Mackill, D. J. (2013) Applying genomics tools for breeding submergence tolerance in rice In Tuberosa A. & Varshney R. (Eds.), Translational genomics for crop breeding (pp. 9–30). Cham, Switzerland: John Wiley & Sons Ltd.

[pld3240-bib-0055] Septiningsih, E. M. , Hidayatun, N. , Sanchez, D. L. , Nugraha, Y. , Carandang, J. , Pamplona, A. M. , … Mackill, D. J. (2015). Accelerating the development of new submergence tolerant rice varieties: The case of Ciherang‐Sub1 and PSB Rc18‐Sub1. Euphytica, 202, 259–268. 10.1007/s10681-014-1287-x

[pld3240-bib-0056] Septiningsih, E. M. , Ignacio, J. C. I. , Sendon, P. M. D. , Sanchez, D. L. , Ismail, A. M. , & Mackill, D. J. (2013). QTL mapping and confirmation for tolerance of anaerobic conditions during germination derived from the rice landrace Ma‐Zhan Red. TAG. Theoretical and Applied Genetics, 126, 1357–1366. 10.1007/s00122-013-2057-1 23417074

[pld3240-bib-0057] Septiningsih, E. M. , & Mackill, D. J. (2018). Genetics and breeding of flooding tolerance in rice In Sasaki T. & Ashikari M. (Eds.), Rice genomics, genetics and breeding (pp. 275–295). Singapore: Springer Singapore.

[pld3240-bib-0058] Septiningsih, E. M. , Pamplona, A. M. , Sanchez, D. L. , Neeraja, C. N. , Vergara, G. V. , Heuer, S. , … Mackill, D. J. (2009). Development of submergence‐tolerant rice cultivars: The Sub1 locus and beyond. Annals of Botany, 103, 151–160. 10.1093/aob/mcn206 18974101 PMC2707316

[pld3240-bib-0059] Septiningsih, E. M. , Sanchez, D. L. , Singh, N. , Sendon, P. M. D. , Pamplona, A. M. , Heuer, S. , & Mackill, D. J. (2012). Identifying novel QTLs for submergence tolerance in rice cultivars IR72 and Madabaru. TAG. Theoretical and Applied Genetics, 124, 867–874. 10.1007/s00122-011-1751-0 22083356

[pld3240-bib-0060] Sharma, N. , Dang, T. M. , Singh, N. , Ruzicic, S. , Mueller‐Roeber, B. , Baumann, U. , & Heuer, S. (2018). Allelic variants of *OsSUB1A* cause differential expression of transcription factor genes in response to submergence in rice. Rice, 11, 2 10.1186/s12284-017-0192-z 29313187 PMC5758481

[pld3240-bib-0061] Shiono, K. , Ando, M. , Nishiuchi, S. , Takahashi, H. , Watanabe, K. , Nakamura, M. , … Kato, K. (2014). RCN1/OsABCG5, an ATP‐binding cassette (ABC) transporter, is required for hypodermal suberization of roots in rice (*Oryza sativa*). The Plant Journal, 80, 40–51. 25041515 10.1111/tpj.12614

[pld3240-bib-0062] Singh, N. , Dang, T. T. M. , Vergara, G. V. , Pandey, D. M. , Sanchez, D. , Neeraja, C. N. , … Heuer, S. (2010). Molecular marker survey and expression analyses of the rice submergence‐tolerance gene *SUB1A* . TAG. Theoretical and Applied Genetics, 121, 1441–1453. 10.1007/s00122-010-1400-z 20652530

[pld3240-bib-0063] Singh, P. , & Sinha, A. K. (2016). A positive feedback loop governed by SUB1A interaction with MITOGEN‐ACTIVATED PROTEIN KINASE3 imparts submergence tolerance in rice. The Plant Cell, 28, 1127–1143. 27081183 10.1105/tpc.15.01001 PMC4904673

[pld3240-bib-0064] Singh, R. , Singh, Y. , Xalaxo, S. , Verulkar, S. , Yadav, N. , Singh, S. , … Singh, N. K. (2016). From QTL to variety‐harnessing the benefits of QTLs for drought, flood and salt tolerance in mega rice varieties of India through a multi‐institutional network. Plant Science, 242, 278–287. 10.1016/j.plantsci.2015.08.008 26566845

[pld3240-bib-0065] Taylor, D. L. (1942). Effects of oxygen on respiration, fermentation and growth in wheat and rice. Science, 95, 129–130. 10.1126/science.95.2457.129 17795659

[pld3240-bib-0066] Toledo, A. M. U. , Ignacio, J. C. I. , Casal, C. , Gonzaga, Z. J. , Mendioro, M. S. , & Septiningsih, E. M. (2015). Development of improved Ciherang‐Sub1 having tolerance to anaerobic germination conditions. Plant Breeding and Biotechnology, 3, 1–11. 10.9787/PBB.2015.3.2.077

[pld3240-bib-0067] Tuong, T. P. , Singh, A. K. , Siopongco, J. D. , & Wade, L. J. (2000). Constraints to high yield of dry‐seeded rice in the rainy season of a humid tropic environment. Plant Production Science, 3, 164–172. 10.1626/pps.3.164

[pld3240-bib-0068] Voesenek, L. A. C. J. , & Bailey‐Serres, J. (2015). Flood adaptive traits and processes: An overview. New Phytologist, 206, 57–73. 10.1111/nph.13209 25580769

[pld3240-bib-0069] Vreeburg, R. A. M. , Benschop, J. J. , Peeters, A. J. M. , Colmer, T. D. , Ammerlaan, A. H. M. , Staal, M. , … Voesenek, L. A. C. J. (2005). Ethylene regulates fast apoplastic acidification and expansin A transcription during submergence‐induced petiole elongation in *Rumex palustris* . The Plant Journal, 43, 597–610. 10.1111/j.1365-313X.2005.02477.x 16098112

[pld3240-bib-0070] Wang, L. , Si, Y. , Dedow, L. K. , Shao, Y. , Liu, P. , & Brutnell, T. P. (2011). A low‐cost library construction protocol and data analysis pipeline for Illumina‐based strand‐specific multiplex RNA‐seq. PLoS One, 6, e26426. 22039485 10.1371/journal.pone.0026426 PMC3198403

[pld3240-bib-0071] Waterworth, W. M. , Bray, C. M. , & West, C. E. (2019). Seeds and the art of genome maintenance. Frontiers in Plant Science, 10, 706 10.3389/fpls.2019.00706 31214224 PMC6554324

[pld3240-bib-0072] Winkel, A. , Colmer, T. D. , Ismail, A. M. , & Pedersen, O. (2013). Internal aeration of paddy field rice (*Oryza sativa*) during complete submergence–importance of light and floodwater O_2_ . New Phytologist, 197(4), 1193–1203. https://doi:10.1111/nph.12048 23215967 10.1111/nph.12048

[pld3240-bib-0073] Winkel, A. , Pedersen, O. , Ella, E. , Ismail, A. M. , & Colmer, T. D. (2014). Gas film retention and underwater photosynthesis during field submergence of four contrasting rice genotypes. Journal of Experimental Botany, 65, 3225–3233. 10.1093/jxb/eru166 24759881 PMC4071835

[pld3240-bib-0074] Xu, K. , Xu, X. , Fukao, T. , Canlas, P. , Maghirang‐Rodriguez, R. , Heuer, S. , … Mackill, D. J. (2006). Sub1A is an ethylene‐response‐factor‐like gene that confers submergence tolerance to rice. Nature, 442, 705–708. 10.1038/nature04920 16900200

[pld3240-bib-0075] Yadav, U. P. , Ivakov, A. , Feil, R. , Duan, G. Y. , Walther, D. , Giavalisco, P. , … Lunn, J. E. (2014). The sucrose‐trehalose 6‐phosphate (Tre6P) nexus: Specificity and mechanisms of sucrose signalling by Tre6P. Journal of Experimental Botany, 65, 1051–1068. 10.1093/jxb/ert457 24420566 PMC3935566

[pld3240-bib-0076] Yamauchi, M. , Aguilar, A. M. , Vaughan, D. A. , & Seshu, D. V. (1993). Rice (*Oryza sativa* L.) germplasm suitable for direct sowing under flooded soil surface. Euphytica, 67, 177–184. 10.1007/BF00040619

[pld3240-bib-0077] Yeung, E. , Bailey‐Serres, J. , & Sasidharan, R. (2019). After the deluge: Plant revival post‐flooding. Trends in Plant Science, 24, 443–454. 10.1016/j.tplants.2019.02.007 30857921

[pld3240-bib-0078] Yeung, E. , van Veen, H. , Vashisht, D. , Sobral Paiva, A. L. , Hummel, M. , Rankenberg, T. , … Sasidharan, R. (2018). A stress recovery signaling network for enhanced flooding tolerance in *Arabidopsis thaliana* . Proceedings of the National Academy of Sciences, 115, E6085–E6094. 10.1073/pnas.1803841115 PMC6042063 29891679

[pld3240-bib-0079] Yu, S. M. , Lee, H. T. , Lo, S. F. , & Ho, T. H. D. (2019). How does rice cope with too little oxygen during its early life? New Phytologist. 10.1111/nph.16395 31880324

[pld3240-bib-0080] Yu, X. , Zhou, L. , Zhang, J. , Yu, H. , Xiong, F. , & Wang, Z. (2015). Comparison of starch granule development and physicochemical properties of starches in wheat pericarp and endosperm. Journal of the Science of Food and Agriculture, 95, 148–157. 10.1002/jsfa.6696 24740388

[pld3240-bib-0081] Zhang, Y. , Primavesi, L. F. , Jhurreea, D. , Andralojc, P. J. , Mitchell, R. A. C. , Powers, S. J. , … Paul, M. J. (2009). Inhibition of SNF1‐related protein kinase1 activity and regulation of metabolic pathways by trehalose‐6‐phosphate. Plant Physiology, 149, 1860–1871. 10.1104/pp.108.133934 19193861 PMC2663748

