# Supplementary figures and images for: Flood resilience loci SUBMERGENCE 1 and ANAEROBIC GERMINATION 1 interact in seedlings established underwater
Source: Plant Direct. 2020 Jul 21;4(7):e00240. doi: 10.1002/pld3.240 (PMC7403837; doi:10.1002/pld3.240)

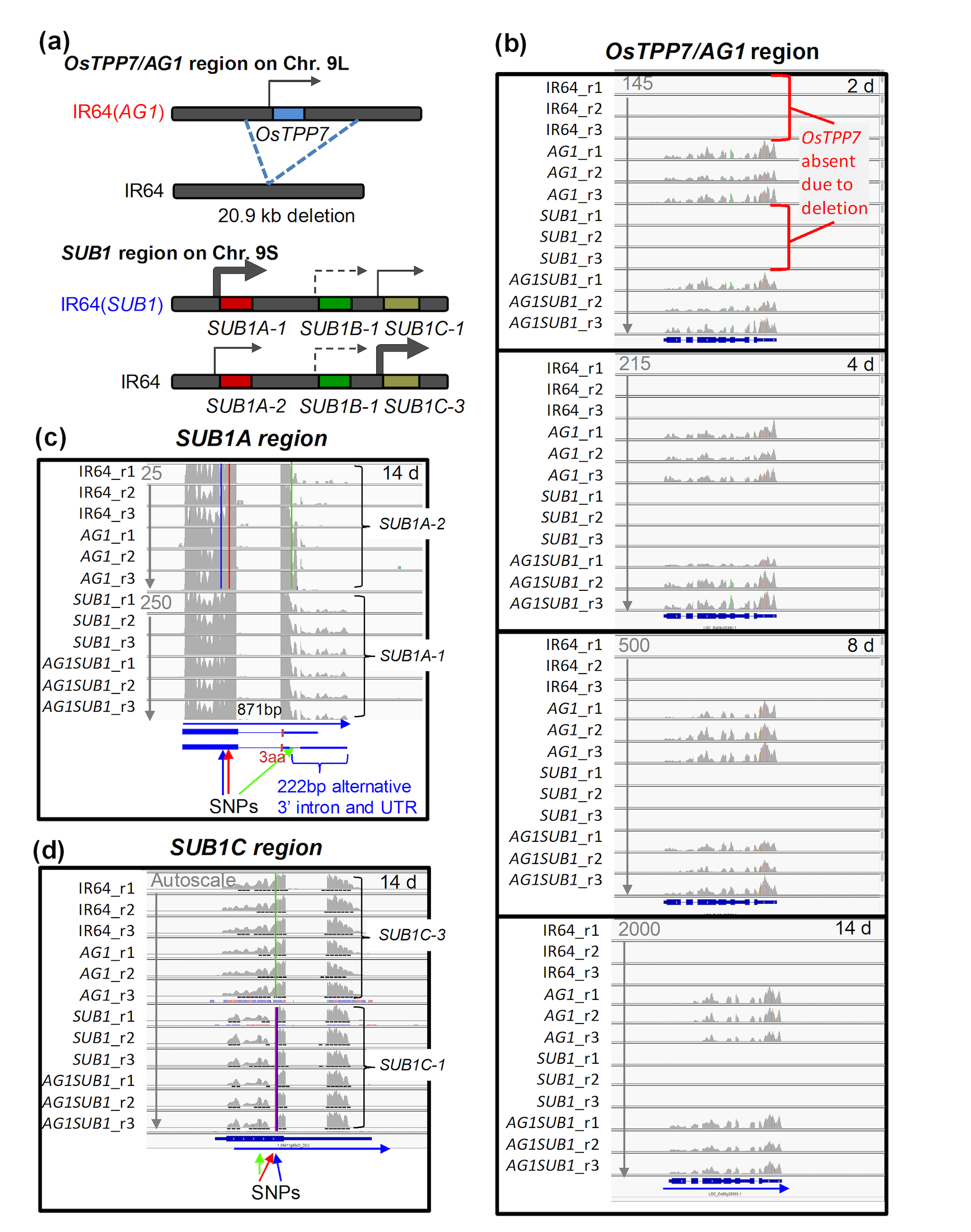

Supplement: Supplementary file 1 — Fig S1 [file PLD3-4-e00240-s001.tiff]

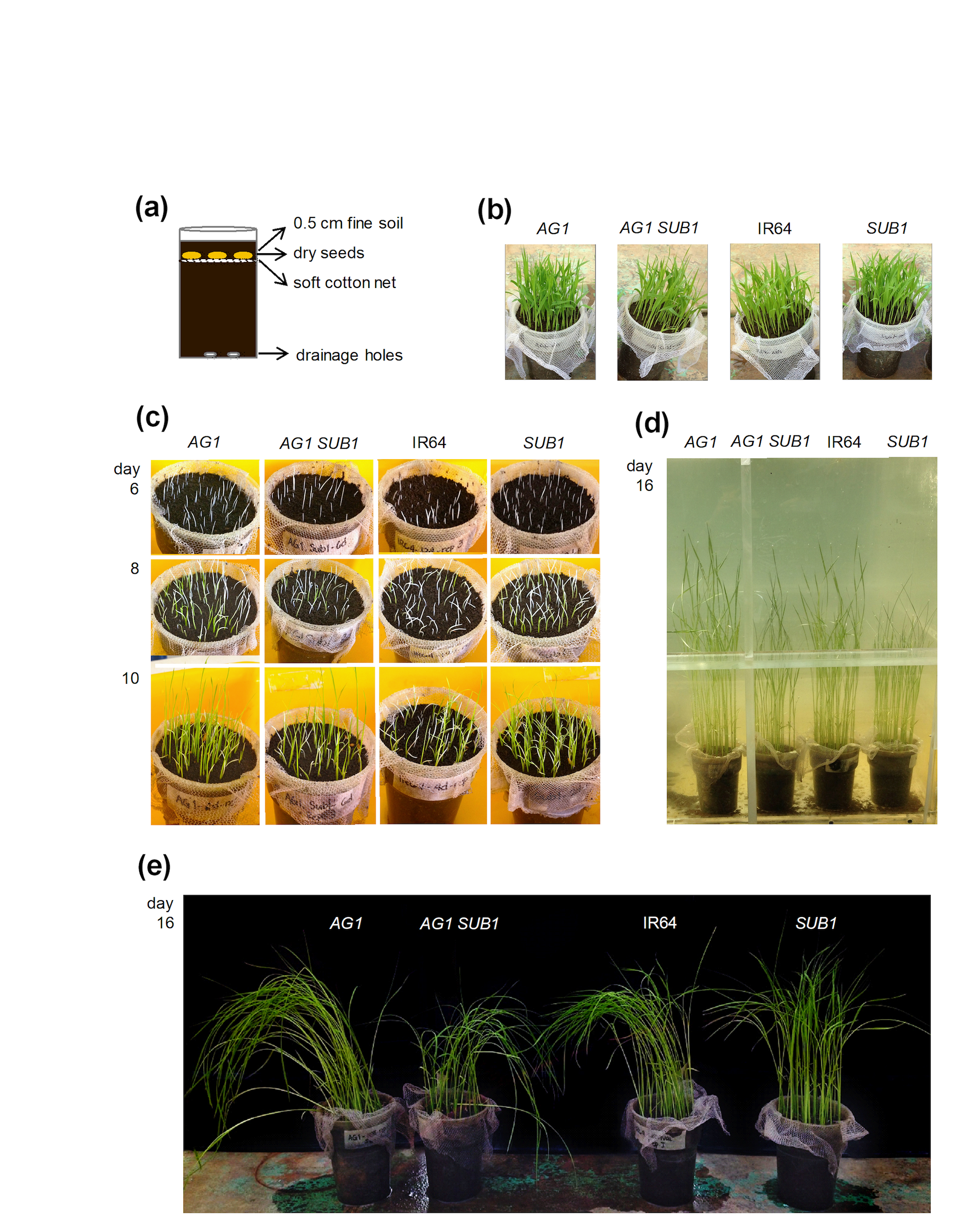

Supplement: Supplementary file 2 — Fig S2 [file PLD3-4-e00240-s002.tiff]

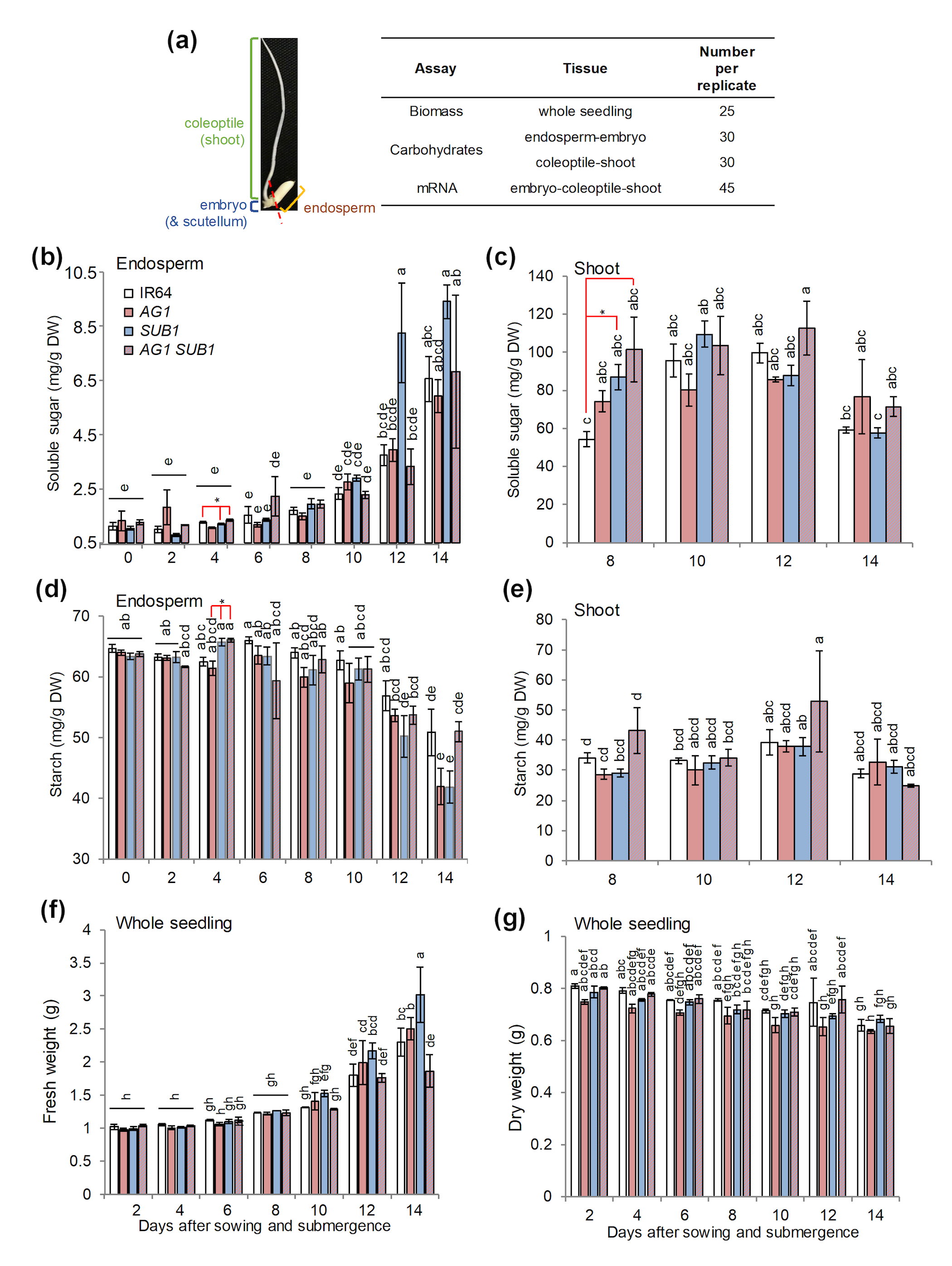

Supplement: Supplementary file 3 — Fig S3 [file PLD3-4-e00240-s003.tiff]

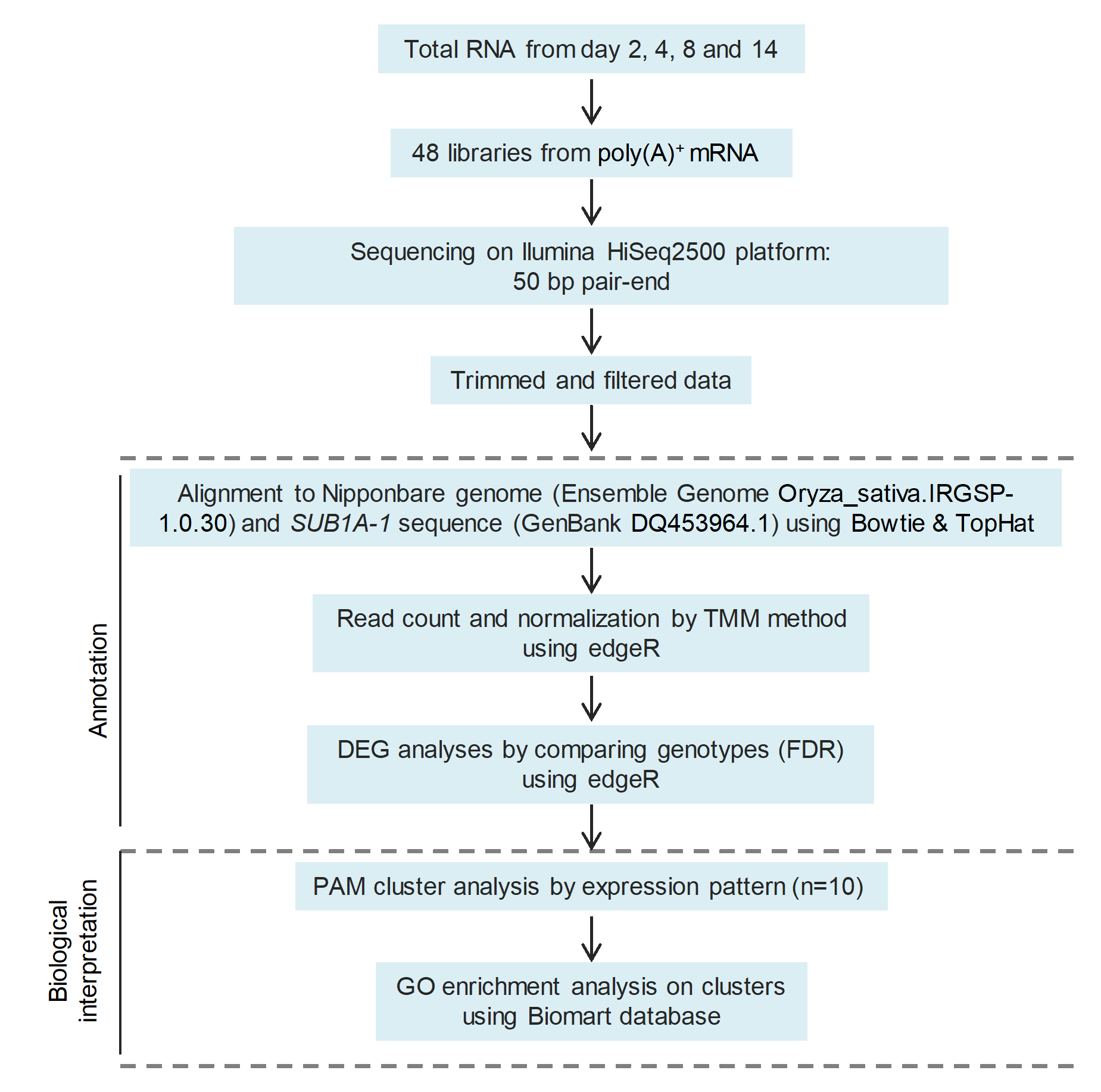

Supplement: Supplementary file 4 — Fig S4 [file PLD3-4-e00240-s004.tif]

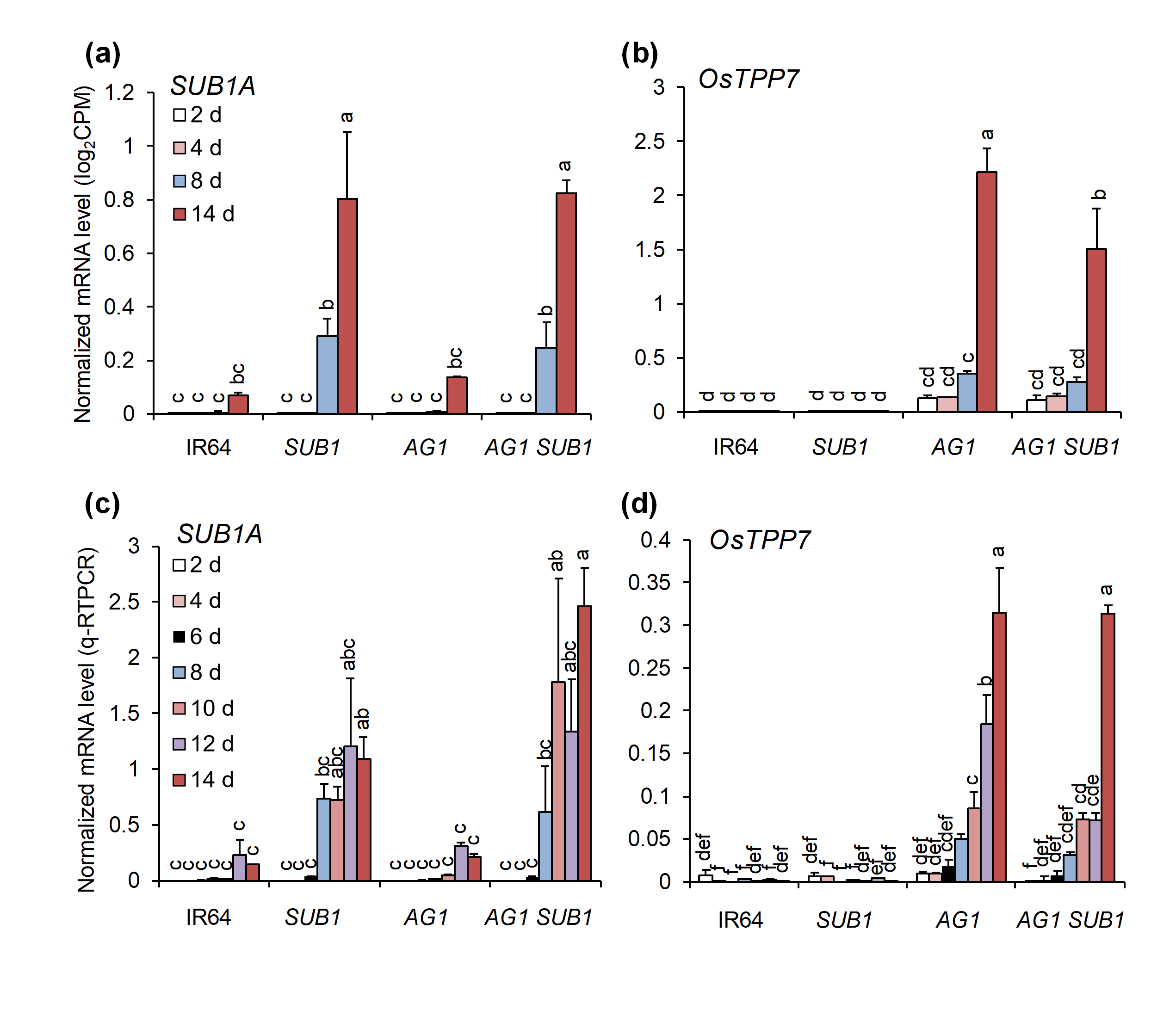

Supplement: Supplementary file 5 — Fig S5 [file PLD3-4-e00240-s005.tif]

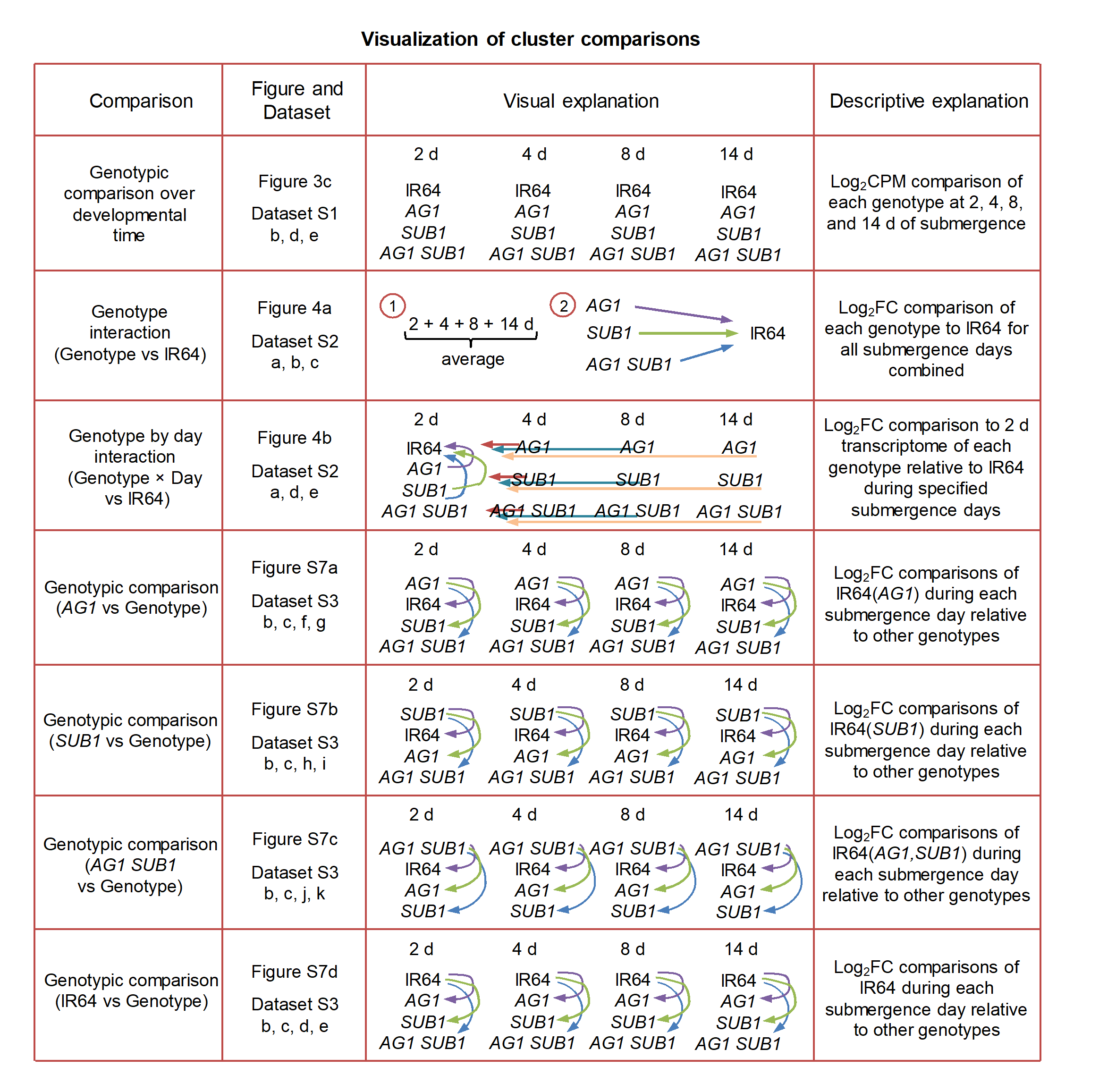

Supplement: Supplementary file 6 — Fig S6 [file PLD3-4-e00240-s006.tif]

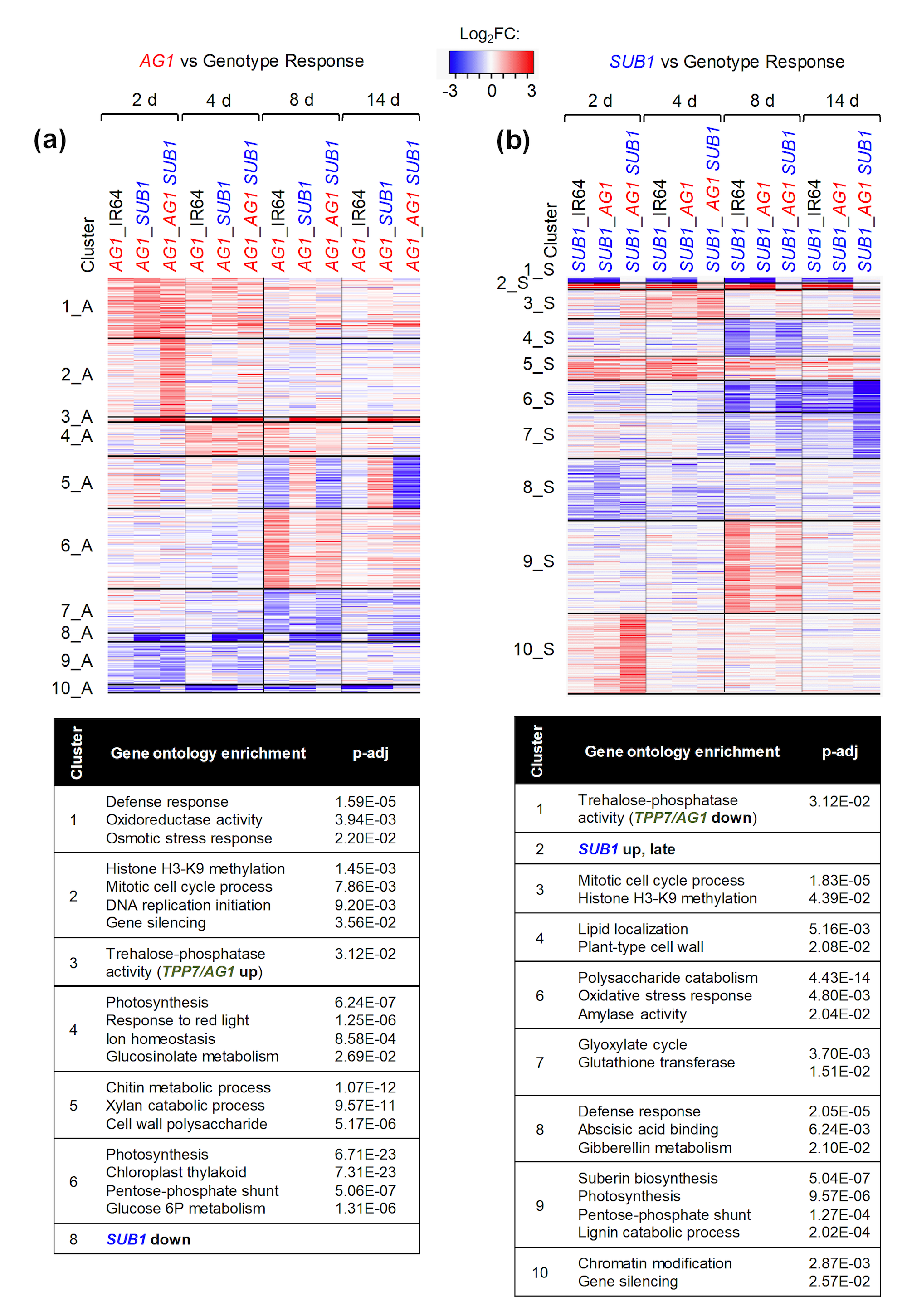

Supplement: Supplementary file 7 — Fig S7a–b [file PLD3-4-e00240-s007.tiff]

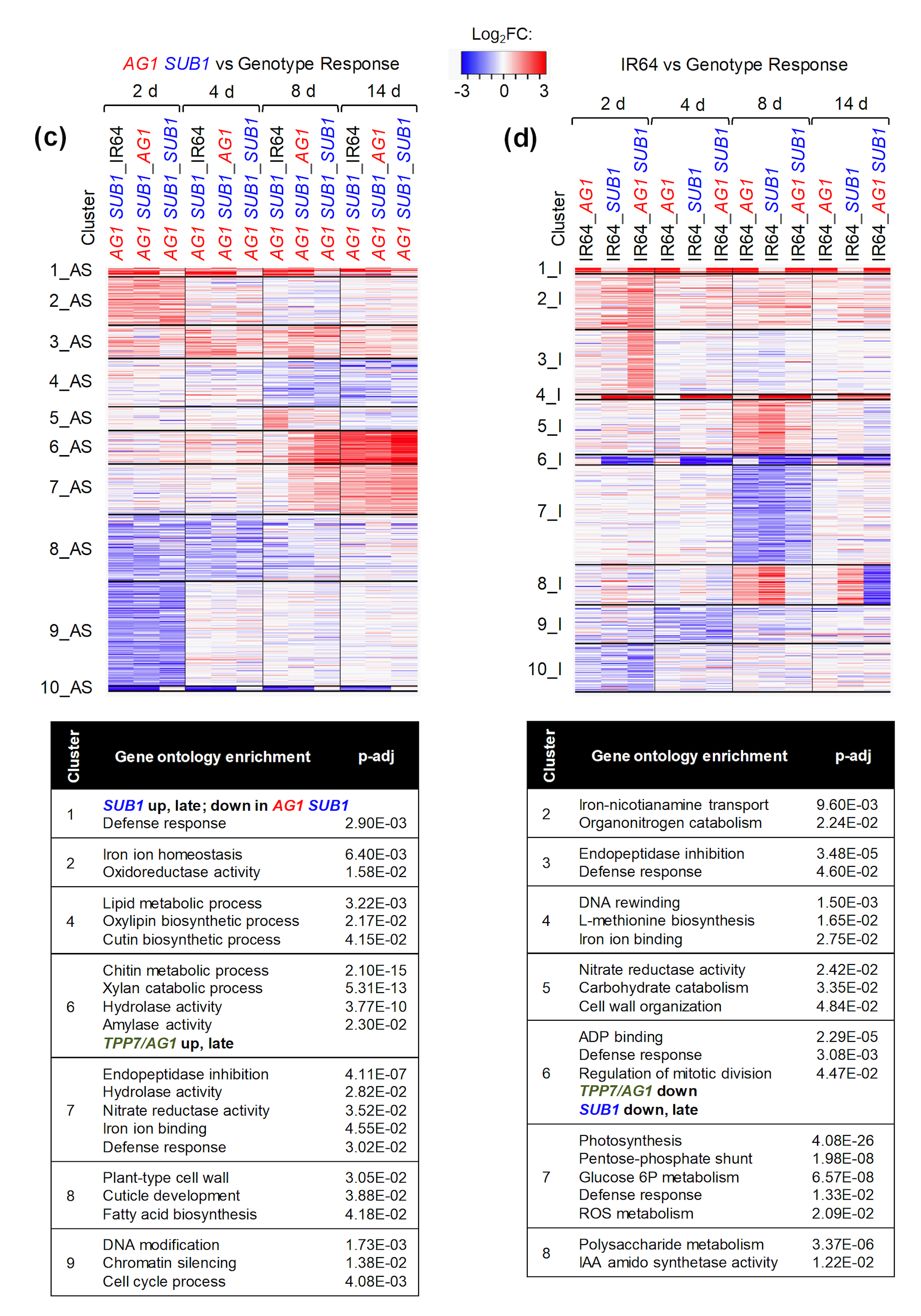

Supplement: Supplementary file 8 — Fig S7C_D [file PLD3-4-e00240-s008.tiff]
