# Supplementary material for: Flood resilience loci SUBMERGENCE 1 and ANAEROBIC GERMINATION 1 interact in seedlings established underwater
Source: Plant Direct. 2020 Jul 21;4(7):e00240. doi: 10.1002/pld3.240 (PMC7403837; doi:10.1002/pld3.240)
Supplement: Supplementary file 14 — Supplementary Material [file PLD3-4-e00240-s014.pdf]

Flood resilience loci *SUBMERGENCE 1* and *ANAEROBIC GERMINATION 1* interact in seedlings established underwater

Rejbana Alam[1*], Maureen Hummel[1*], Elaine Yeung[1*], Anna M. Locke[1,3], John Carlos I. Ignacio[2], Miriam D. Baltazar[4], Zhenyu Jia[1], Abdelbagi M. Ismail[2], Endang M. Septiningsih[2,5] and Julia Bailey-Serres[1]

[*] co-contributing authors

Supplemental Figures and Legends

Figure S1. *AG1* and *SUB1* alleles/haplotypes used in this study. (a) Representation of the chromosome 9 regions encoding *TPP7*/*AG1* from KHO and *SUB1A-1* from FR13A (Kretzschmar et al., 2015; Xu et al., 2006). *AG1* was introgressed into IR64 to generate IR64(A*G1*) (Kretzschmar et al., 2015), and *SUB1A-1* was introgressed into IR64 to generate IR64(*SUB1*). IR64 lacks *AG1*. IR64 encodes *SUB1A-2*, which produces the *SUB1* transcript at lower levels than the *SUB1A-1* allele from FR13A. The paralogous genes *SUB1B* and *SUB1C* produce transcripts at different levels in the two haplotypes represented, but submergence tolerance is determined by *SUB1A-1* (Fukao et al., 2006, 2009; Xu et al., 2006). (b) RNA-seq analysis of the *AG1* region from 3 bioreplicates (r1, r2, r3) at the submergence time points assayed by transcriptomics for the 4 genotypes. Full experimental analysis presented later in the Results and Discussion. The data confirm that IR64(*AG1*) and IR64(*AG1,SUB1*) encode *TPP7,* whereas IR64 and IR64(*SUB1*) lack this gene*.* (c-d) RNA-seq analysis of the *SUB1A* and *SUB1C* regions in day 14 transcriptomes of submerged shoots of the four genotypes. Read mapping was to *SUB1A-1* and SUB1C-3 of IR64. Nucleotide polymorphisms confirm that IR64 and IR64(*AG1*) encode *SUB1A-2* and *SUB1C-3* (Singh et al., 2010), whereas IR64(*SUB1*) and IR64(*AG1,SUB1*) encode *SUB1A-1* and *SUB1C-1* from FR13A (Xu et al., 2006). Data highlight an alternative 3' intron within the 3' untranslated region of SUB1A that accumlates to higher levels in *SUB1A-1*.

Figure S2. Rice establishment by dry seeding followed by prolonged submergence is influenced by *AG1* and *SUB1*. (a) Schematic diagram of the experimental set-up of dry seeding and continual submergence. Dry seeds were sown directly into pots at a soil depth of 0.5 cm on top of a soft cotton net. Pots had small drainage holes at the bottom with 11 cm depth of fine soil covered with the net. (b) Control plants photographed after 7 d of growth in normal air conditions in a nethouse under ambient light. (c) Representative photographs of seedlings immediately after desubmergence at 6, 8, and 10 d of submergence. (d) Submerged plants photographed at 16 d of

submergence in the aquaria. (e) Photo of desubmerged plants after 16 d of submergence. Photos are representative of 3 experimental replicates.

Figure S3. Visualization of harvested tissues, soluble sugar, and starch contents of the endosperm and shoot, and seedling biomass of plants grown under continual submergence. IR64, IR64(*AG1*), IR64(*SUB1*), and IR64(*AG1,SUB1*) were dry seeded and maintained under constant complete submergence for up to 14 d. (a) Visualization and lists of tissues harvested for each assay. (b) Soluble sugar of endosperm-scutellar-embryo tissue from 0 d [dry seed] to 14 d. (c) Soluble sugar of coleoptile-shoot tissue (excluding seed tissue) at 8, 10, 12, and 14 d of submergence. (d) Starch content of endosperm-scutellar-embryo tissue and (e) shoot tissue. Soluble sugar and starch contents of shoots could not be measured from 0-6 d due to the unavailability of sufficient shoot tissue at these time points. (f) Fresh and (g) dry weight of the whole seedling (including the caryopsis) from 2 to 14 d. All data represent mean ± SE of 3 replicates (n=25-30 seedlings of each genotype per replicate). Different letters are significantly different across all days ($P < 0.05$, ANOVA with Tukey HSD test). Red bars and asterisks indicate significance in pairwise analyses per day.

Figure S4. Flowchart of the mRNA-sequencing and transcriptome analyses.

Figure S5. Allelic variation in temporal regulation of *AG1* and *SUB1A* transcript accumulation. Comparison of *TPP7* and *SUB1A* transcript levels in the genotypes IR64, IR64(*AG1*), IR64(*SUB1*), and IR64(*AG1,SUB1*) monitored by (a-b) RNA-seq, based on mean $\log_2$ counts per million reads (CPM) of transcripts, and (c-d) qRT-PCR. For both, values are normalized to LOC_Os01g53520, observed to be near-constitutive across the time points sampled. Data are mean ± SD from 2 to 3 bioreplicates for RNA-seq and qRT-PCR. Letters indicate significant differences ($P < 0.05$, ANOVA with Tukey HSD test).

Figure S6. Overview of the cluster comparisons in Figure 3, Figure 4, and Figure S7. Arrows indicate the genotype and time point comparisons.

Figure S7. Transcriptomic comparisons between genotypes during seedling establishment under prolonged submergence. (a-d) PAM clustering of genotypic comparisons during 2, 4, 8, and 14 d of submergence for (a) IR64(*AG1*), (b) IR64(*SUB1*), (c) IR64(*AG1,SUB1*), and (d) IR64 relative to the other three genotypes. Genotypes in comparison are separated by an underscore ("_"). Data values for the single genotype used for comparision were the numerator and data values for the three genotypes were in the denominator of the fold change calculation. All clusters are composed of mean $\log_2$FC values of DEGs ($\log_2$FC > |1|; FDR < 0.05). Representative GO

enrichment of notable clusters and genes are listed. More DEGs, log_2FC values, and GO term associations are in Dataset S3.

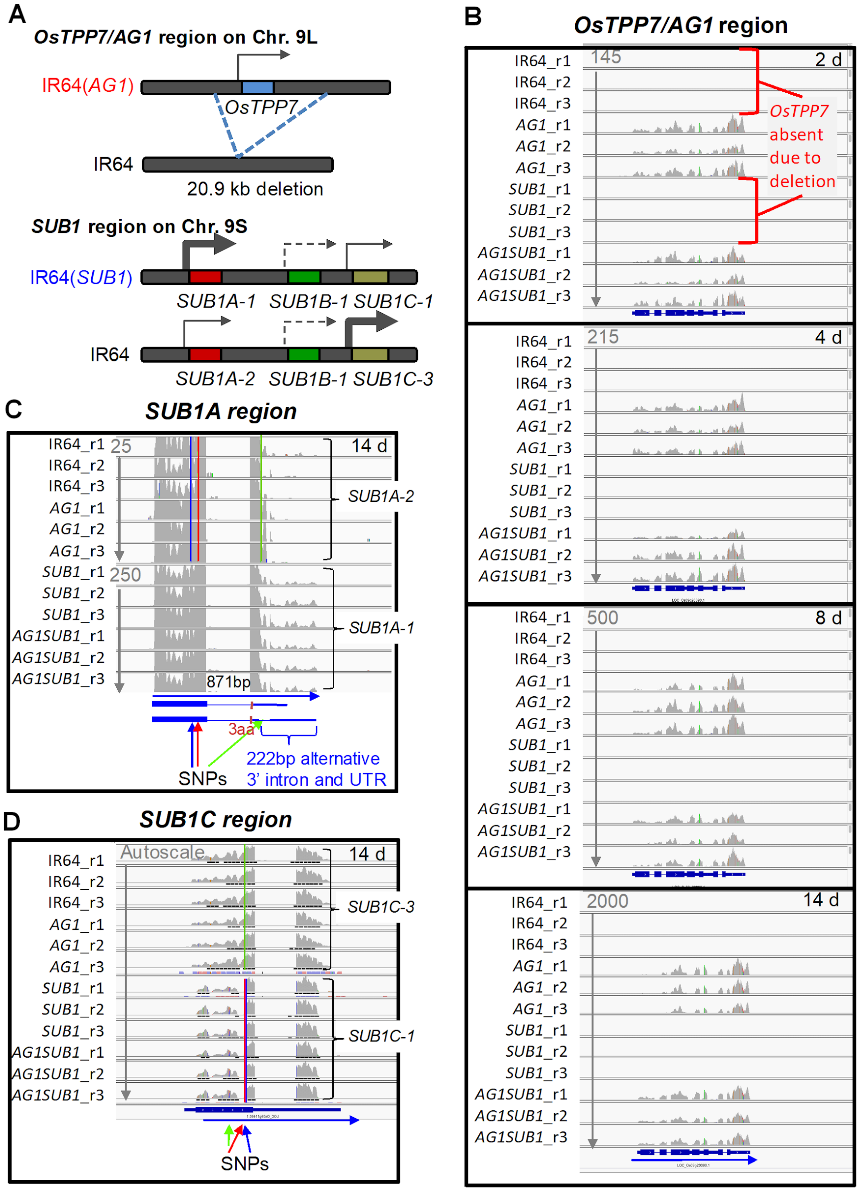

Figure S1. *AG1* and *SUB1* alleles/haplotypes used in this study. (a) Representation of the chromosome 9 regions encoding *TPP7/AG1* from KHO and *SUB1A-1* from FR13A. *AG1* was introgressed into IR64 to generate IR64(*AG1*) (Kretzschmar et al., 2015), and *SUB1A-1* was introgressed into IR64 to generate IR64(*SUB1*). The *SUB1* region haplotype of IR64 encodes *SUB1A-2*, which produces the *SUB1* transcript at lower levels than the *SUB1A-1* allele from FR13A. The paralogs *SUB1B* and *SUB1C* produce transcripts at different levels in the two haplotypes represented (Fukao et al., 2006, 2009; Xu et al., 2006), but submergence tolerance is solely determined by *SUB1A-1* (b) RNA-seq analysis of the *AG1* region from 3 bioreplicates (r1, r2, r3) at the submergence time points assayed for the 4 genotypes. IR64(*AG1*) and IR64(*AG1,SUB1*) encode *TPP7,* whereas IR64 and IR64(*SUB1*) lack this gene. (c-d) RNA-seq analysis of the *SUB1A* and *SUB1C* regions. The genotypes IR64 and IR64(*AG1*) encode *SUB1A-2* and *SUB1C-3*, therefore having low submergence tolerance. IR64(*SUB1*) and IR64(*AG1,SUB1*) encode *SUB1A-1* and *SUB1C-1* from the submergence tolerant donor FR13A. *AG1* was validated by *TPP7* presence. *SUB1A* alleles were confirmed by single nucleotide polymorphism distinctions in the linked *SUB1A-1* and *SUB1C-1* loci in *SUB1*-containing lines (FR13A) and *SUB1A-2* and *SUB1C-3* loci of IR64 (Singh et al., 2010).

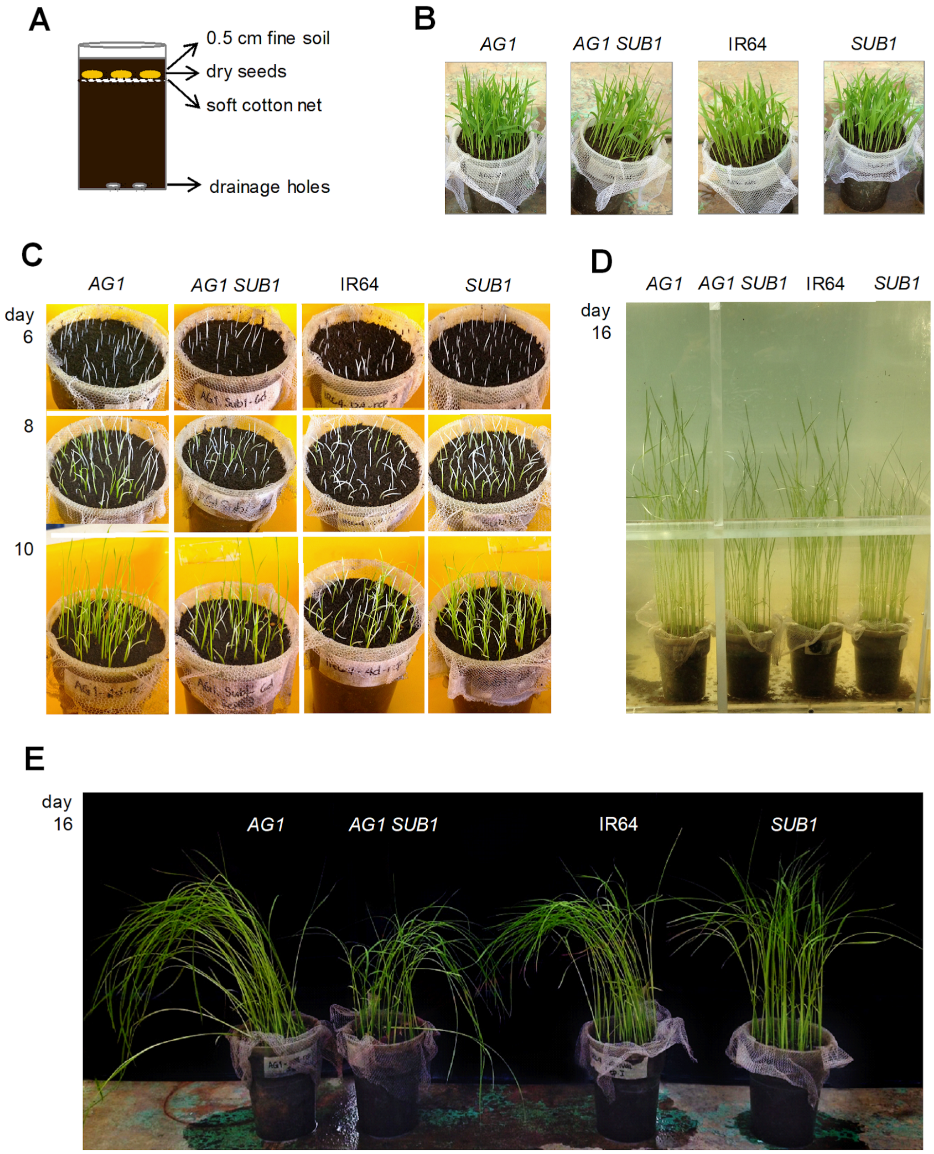

Figure S2. Rice establishment by dry seeding followed by prolonged submergence is influenced by *AG1* and *SUB1*. (a) Schematic diagram of the experimental set-up of dry seeding and continual submergence. Dry seeds were sown directly into pots at a soil depth of 0.5 cm on top of a soft cotton net. Pots had small drainage holes at the bottom with 11 cm depth of fine soil covered with the net. (b) Control plants photographed after 7 d of growth in normal air conditions. (c) Representative photographs of seedlings immediately after desubmergence at 6, 8, and 10 d of submergence. (d) Submerged plants photographed at 16 d of submergence in the aquaria. (e) Photo of desubmerged plants after 16 d of submergence. Photos are representative of 3 experimental replicates.

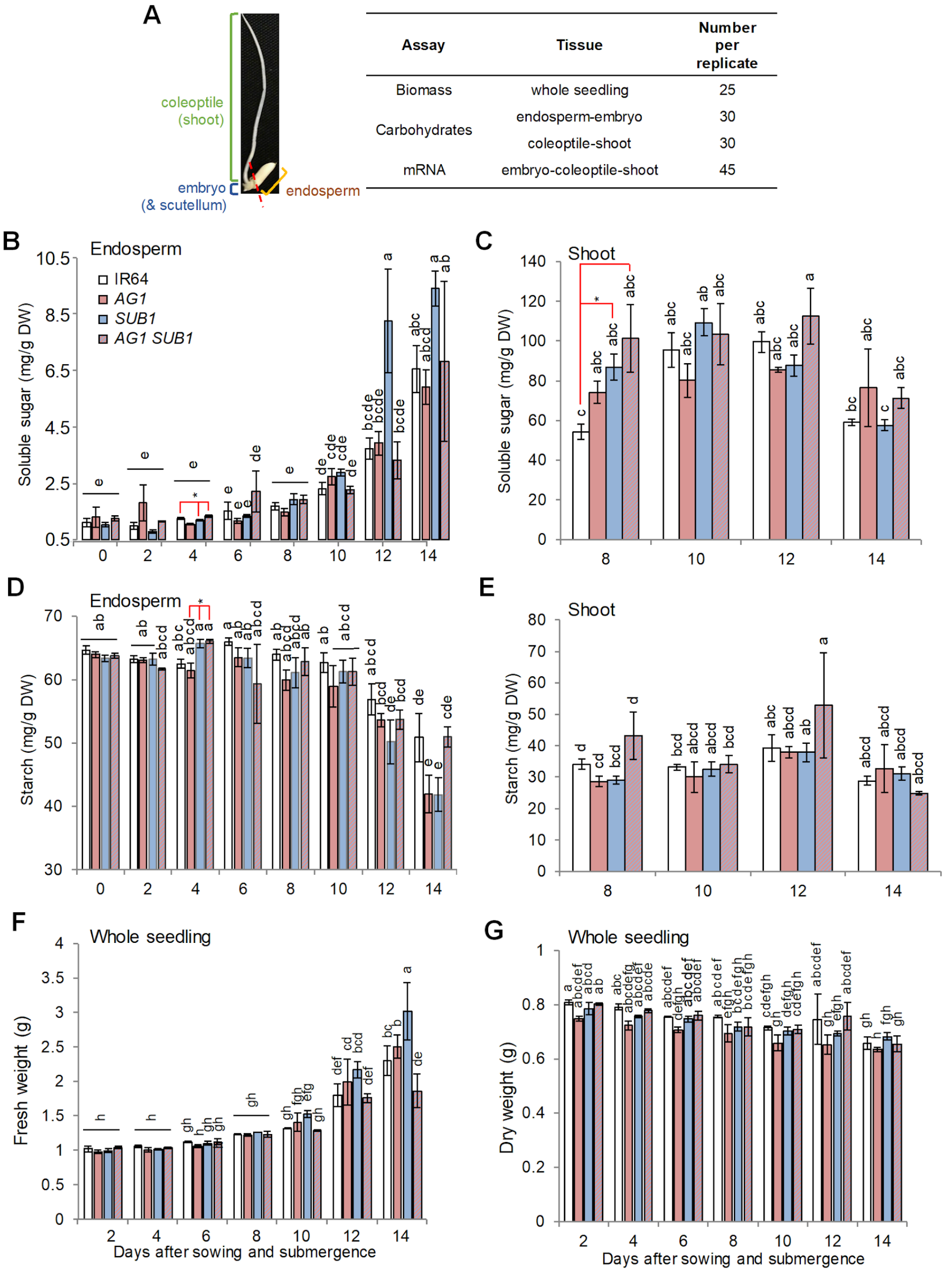

Figure S3. Visualization of harvested tissues, soluble sugar, and starch contents of the endosperm and shoot, and seedling biomass of plants grown under continual submergence. IR64, IR64(*AG1*), IR64(*SUB1*), and IR64(*AG1,SUB1*) were dry seeded and maintained under constant complete submergence for up to 14 d. (a) Visualization and lists of tissues harvested for each assay. (b) Soluble sugar of endosperm-scutellar-embryo tissue from 0 d [dry seed] to 14 d. (c) Soluble sugar of coleoptile-shoot tissue (excluding seed tissue) at 8, 10, 12, and 14 d of submergence. (d) Starch content of endosperm-scutellar-embryo tissue and (e) shoot tissue. Soluble sugar and starch contents of shoots could not be measured from 0-6 d due to the unavailability of sufficient shoot tissue at these time points. (f) Fresh and (g) dry weight of the whole seedling (including the caryopsis) from 2 to 14 d. All data represent mean ± SE of 3 replicates (n=25-30 seedlings of each genotype per replicate). Different letters are significantly different across all days ($P < 0.05$, ANOVA with Tukey HSD test). Red bars and asterisks indicate significance in pairwise analyses per day.

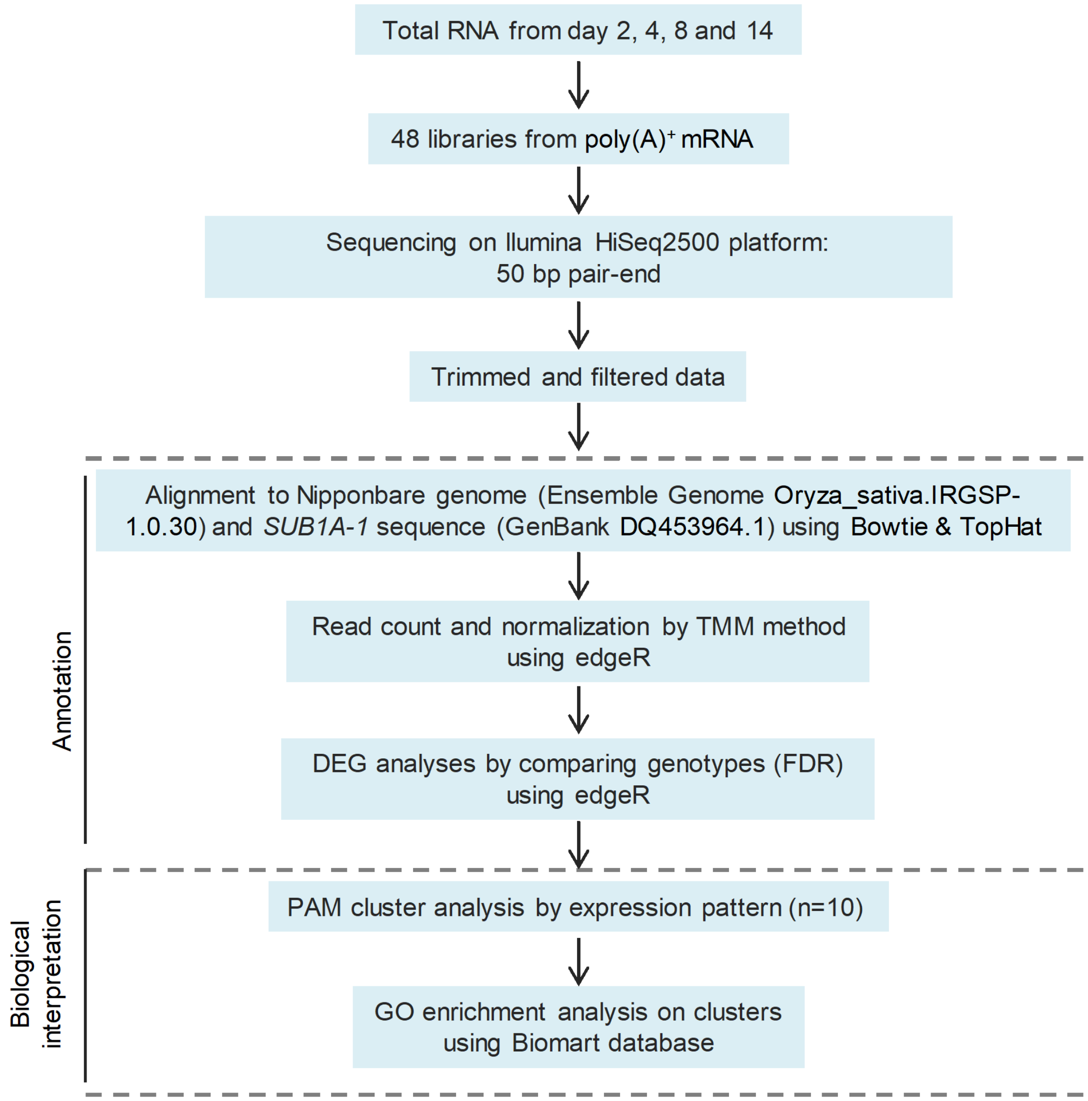

Figure S4. Flowchart of the mRNA-sequencing and transcriptome analyses.

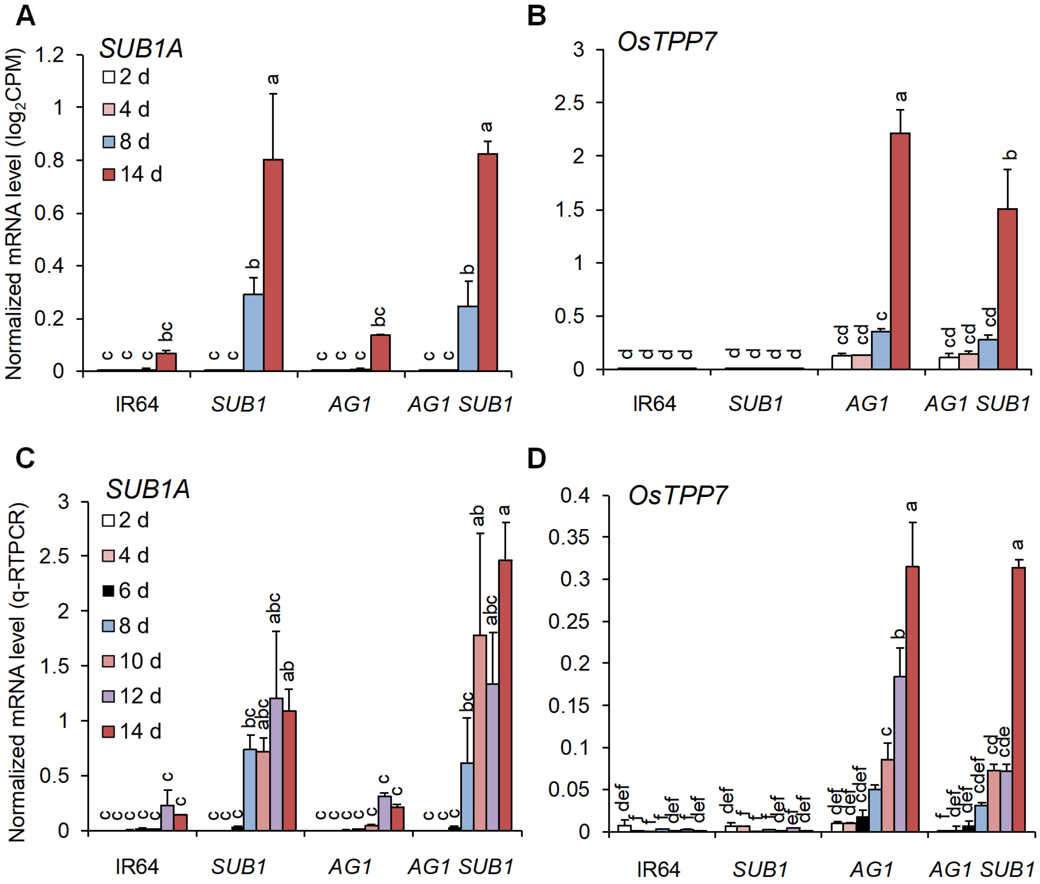

Figure S5. Allelic variation in temporal regulation of *AG1* and *SUB1A* transcript accumulation. Comparison of *TPP7* and *SUB1A* transcript levels in the genotypes IR64, IR64(*AG1*), IR64(*SUB1*), and IR64(*AG1,SUB1*) monitored by (a-b) RNA-seq, based on mean $\log_2$ counts per million reads (CPM) of transcripts, and (c-d) qRT-PCR. For both, values are normalized to LOC_Os01g53520, observed to be near-constitutive across the time points sampled. Data are mean ± SD from 2 to 3 bioreplicates for RNA-seq and qRT-PCR. Letters indicate significant differences ($P < 0.05$, ANOVA with Tukey HSD test).

Visualization of cluster comparisons

Comparison	Figure and Dataset	Visual explanation	Descriptive explanation
Genotypic comparison over developmental time	Fig. 3C<br>Supplemental Dataset S1, B, D and E	2 d 4 d 8 d 14 d<br>IR64 IR64 IR64 IR64<br>*AG1* *AG1* *AG1* *AG1*<br>*SUB1* *SUB1* *SUB1* *SUB1*<br>*AG1 SUB1* *AG1 SUB1* *AG1 SUB1* *AG1 SUB1*	Log_2CPM comparison of each genotype at 2, 4, 8, and 14 d of submergence
Genotype interaction (Genotype vs IR64)	Fig. 4A<br>Supplemental Dataset S2, A, B and C	① 2 + 4 + 8 + 14 d average<br>② *AG1* → IR64; *SUB1* → IR64; *AG1 SUB1* → IR64	Log_2FC comparison of each genotype to IR64 for all submergence days combined
Genotype by day interaction (Genotype × Day vs IR64)	Fig. 4B<br>Supplemental Dataset S2, A, D and E	2 d 4 d 8 d 14 d<br>IR64 *AG1* *AG1* *AG1*<br>*AG1* *SUB1* *SUB1* *SUB1*<br>*SUB1*<br>*AG1 SUB1* *AG1 SUB1* *AG1 SUB1* *AG1 SUB1*	Log_2FC comparison to 2 d transcriptome of each genotype relative to IR64 during specified submergence days
Genotypic comparison (*AG1* vs Genotype)	Supplemental Fig. S7A<br>Supplemental Dataset S3 B, C, F and G	2 d 4 d 8 d 14 d<br>*AG1* *AG1* *AG1* *AG1*<br>IR64 IR64 IR64 IR64<br>*SUB1* *SUB1* *SUB1* *SUB1*<br>*AG1 SUB1* *AG1 SUB1* *AG1 SUB1* *AG1 SUB1*	Log_2FC comparisons of IR64(*AG1*) during each submergence day relative to other genotypes
Genotypic comparison (*SUB1* vs Genotype)	Supplemental Fig. S7B<br>Supplemental Dataset S3 B, C, H and I	2 d 4 d 8 d 14 d<br>*SUB1* *SUB1* *SUB1* *SUB1*<br>IR64 IR64 IR64 IR64<br>*AG1* *AG1* *AG1* *AG1*<br>*AG1 SUB1* *AG1 SUB1* *AG1 SUB1* *AG1 SUB1*	Log_2FC comparisons of IR64(*SUB1*) during each submergence day relative to other genotypes
Genotypic comparison (*AG1 SUB1* vs Genotype)	Supplemental Fig. S7C<br>Supplemental Dataset S3 B, C, J and K	2 d 4 d 8 d 14 d<br>*AG1 SUB1* *AG1 SUB1* *AG1 SUB1* *AG1 SUB1*<br>IR64 IR64 IR64 IR64<br>*AG1* *AG1* *AG1* *AG1*<br>*SUB1* *SUB1* *SUB1* *SUB1*	Log_2FC comparisons of IR64(*AG1*,*SUB1*) during each submergence day relative to other genotypes
Genotypic comparison (IR64 vs Genotype)	Supplemental Fig. S7D<br>Supplemental Dataset S3 B, C, D and E	2 d 4 d 8 d 14 d<br>IR64 IR64 IR64 IR64<br>*AG1* *AG1* *AG1* *AG1*<br>*SUB1* *SUB1* *SUB1* *SUB1*<br>*AG1 SUB1* *AG1 SUB1* *AG1 SUB1* *AG1 SUB1*	Log_2FC comparisons of IR64 during each submergence day relative to other genotypes

Figure S6. Overview of the cluster comparisons in Figure 3, Figure 4, and Figure S7. Arrows indicate the genotype and time point comparisons.

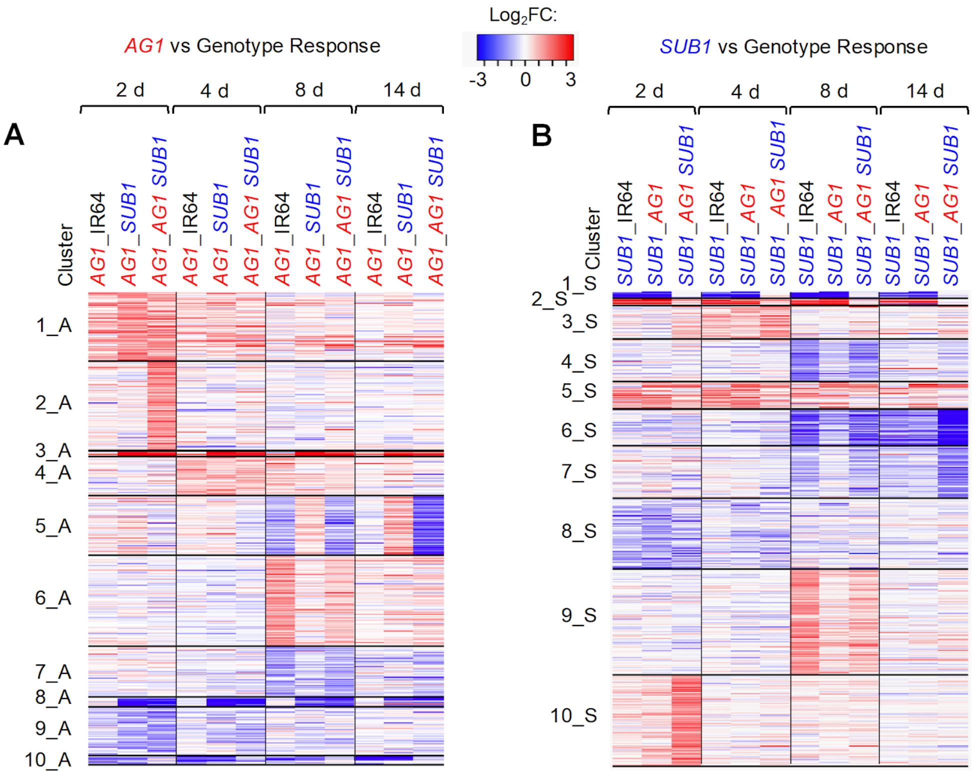

Cluster	Gene ontology enrichment	p-adj
1	Defense response<br>Oxidoreductase activity<br>Osmotic stress response	1.59E-05<br>3.94E-03<br>2.20E-02
2	Histone H3-K9 methylation<br>Mitotic cell cycle process<br>DNA replication initiation<br>Gene silencing	1.45E-03<br>7.86E-03<br>9.20E-03<br>3.56E-02
3	Trehalose-phosphatase activity (***TPP7/AG1* up**)	3.12E-02
4	Photosynthesis<br>Response to red light<br>Ion homeostasis<br>Glucosinolate metabolism	6.24E-07<br>1.25E-06<br>8.58E-04<br>2.69E-02
5	Chitin metabolic process<br>Xylan catabolic process<br>Cell wall polysaccharide	1.07E-12<br>9.57E-11<br>5.17E-06
6	Photosynthesis<br>Chloroplast thylakoid<br>Pentose-phosphate shunt<br>Glucose 6P metabolism	6.71E-23<br>7.31E-23<br>5.06E-07<br>1.31E-06
8	***SUB1* down**	

Cluster	Gene ontology enrichment	p-adj
1	Trehalose-phosphatase activity (***TPP7/AG1* down**)	3.12E-02
2	***SUB1* up, late**	
3	Mitotic cell cycle process<br>Histone H3-K9 methylation	1.83E-05<br>4.39E-02
4	Lipid localization<br>Plant-type cell wall	5.16E-03<br>2.08E-02
6	Polysaccharide catabolism<br>Oxidative stress response<br>Amylase activity	4.43E-14<br>4.80E-03<br>2.04E-02
7	Glyoxylate cycle<br>Glutathione transferase	3.70E-03<br>1.51E-02
8	Defense response<br>Abscisic acid binding<br>Gibberellin metabolism	2.05E-05<br>6.24E-03<br>2.10E-02
9	Suberin biosynthesis<br>Photosynthesis<br>Pentose-phosphate shunt<br>Lignin catabolic process	5.04E-07<br>9.57E-06<br>1.27E-04<br>2.02E-04
10	Chromatin modification<br>Gene silencing	2.87E-03<br>2.57E-02

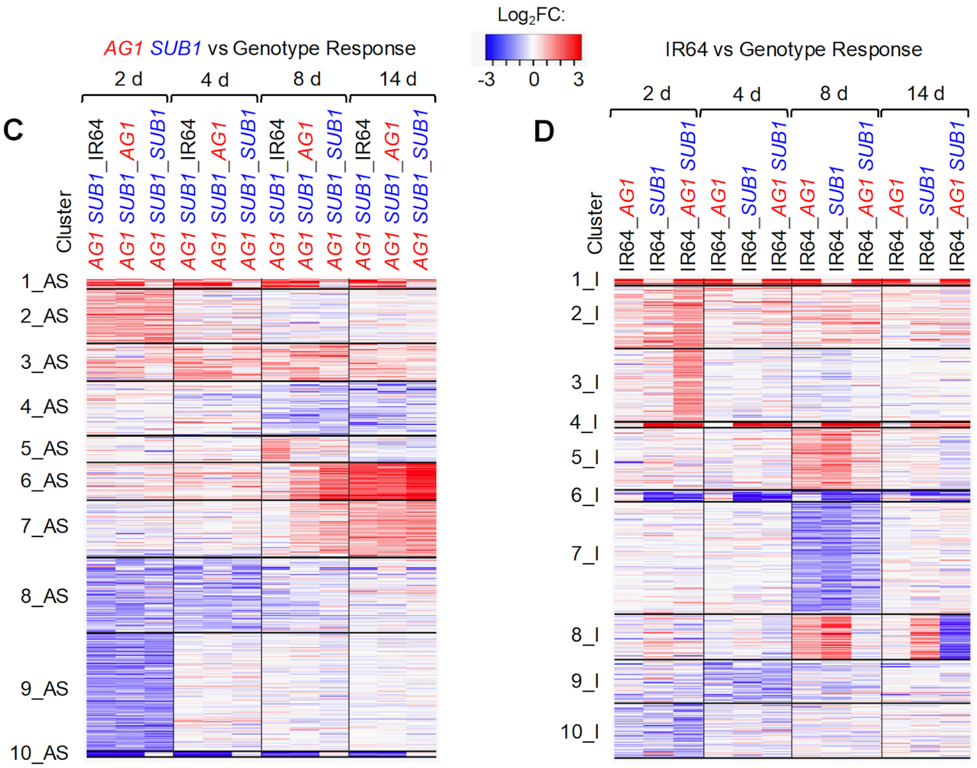

Cluster	Gene ontology enrichment	p-adj
1	***SUB1* up, late; down in *AG1 SUB1***<br>Defense response	 2.90E-03
2	Iron ion homeostasis<br>Oxidoreductase activity	6.40E-03<br>1.58E-02
4	Lipid metabolic process<br>Oxylipin biosynthetic process<br>Cutin biosynthetic process	3.22E-03<br>2.17E-02<br>4.15E-02
6	Chitin metabolic process<br>Xylan catabolic process<br>Hydrolase activity<br>Amylase activity<br>***TPP7/AG1* up, late**	2.10E-15<br>5.31E-13<br>3.77E-10<br>2.30E-02
7	Endopeptidase inhibition<br>Hydrolase activity<br>Nitrate reductase activity<br>Iron ion binding<br>Defense response	4.11E-07<br>2.82E-02<br>3.52E-02<br>4.55E-02<br>3.02E-02
8	Plant-type cell wall<br>Cuticle development<br>Fatty acid biosynthesis	3.05E-02<br>3.88E-02<br>4.18E-02
9	DNA modification<br>Chromatin silencing<br>Cell cycle process	1.73E-03<br>1.38E-02<br>4.08E-03

Cluster	Gene ontology enrichment	p-adj
2	Iron-nicotianamine transport<br>Organonitrogen catabolism	9.60E-03<br>2.24E-02
3	Endopeptidase inhibition<br>Defense response	3.48E-05<br>4.60E-02
4	DNA rewinding<br>L-methionine biosynthesis<br>Iron ion binding	1.50E-03<br>1.65E-02<br>2.75E-02
5	Nitrate reductase activity<br>Carbohydrate catabolism<br>Cell wall organization	2.42E-02<br>3.35E-02<br>4.84E-02
6	ADP binding<br>Defense response<br>Regulation of mitotic division<br>***TPP7/AG1* down**<br>***SUB1* down, late**	2.29E-05<br>3.08E-03<br>4.47E-02
7	Photosynthesis<br>Pentose-phosphate shunt<br>Glucose 6P metabolism<br>Defense response<br>ROS metabolism	4.08E-26<br>1.98E-08<br>6.57E-08<br>1.33E-02<br>2.09E-02
8	Polysaccharide metabolism<br>IAA amido synthetase activity	3.37E-06<br>1.22E-02

Figure S7. Transcriptomic comparisons between genotypes during seedling establishment under prolonged submergence. (a-d) PAM clustering of genotypic comparisons during 2, 4, 8, and 14 d of submergence for (a) IR64(*AG1*), (b) IR64(*SUB1*), (c) IR64(*AG1,SUB1*), and (d) IR64 relative to the other three genotypes. Genotypes in comparison are separated by an underscore ("_"). All clusters composed of mean log_2FC values of DEGs. Representative GO enrichment of notable clusters and genes are listed. More DEGs, log_2FC values, and GO term associations are in Dataset S3.
